# The Oncogenic MicroRNA Hsa-miR-155-5p Targets the Transcription Factor ELK3 and Links It to the Hypoxia Response

**DOI:** 10.1371/journal.pone.0113050

**Published:** 2014-11-17

**Authors:** E. Douglas Robertson, Christine Wasylyk, Tao Ye, Alain C. Jung, Bohdan Wasylyk

**Affiliations:** 1 Institut de Génétique et de Biologie Moléculaire et Cellulaire, Illkirch, France; 2 Centre National de la Recherche Scientifique, UMR7104, Illkirch, France; 3 Institut National de la Santé et de la Recherche Médicale, U964, Illkirch, France; 4 Université de Strasbourg, Illkirch, France; 5 Laboratoire de Biologie Tumorale, Centre Régional de Lutte Contre le Cancer Paul Strauss, EA3430 de l’Université de Strasbourg, Strasbourg, France; Osaka University, Japan

## Abstract

The molecular response to hypoxia is a critical cellular process implicated in cancer, and a target for drug development. The activity of the major player, HIF1α, is regulated at different levels by various factors, including the transcription factor ELK3. The molecular mechanisms of this intimate connection remain largely unknown. Whilst investigating global ELK3-chromatin interactions, we uncovered an unexpected connection that involves the microRNA hsa-miR-155-5p, a hypoxia-inducible oncomir that targets HIF1α. One of the ELK3 chromatin binding sites, detected by Chromatin Immuno-Precipitation Sequencing (ChIP-seq) of normal Human Umbilical Vein Endothelial Cells (HUVEC), is located at the transcription start site of the MIR155HG genes that expresses hsa-miR-155-5p. We confirmed that ELK3 binds to this promoter by ChIP and quantitative polymerase chain reaction (QPCR). We showed that ELK3 and hsa-miR-155-5p form a double-negative regulatory loop, in that ELK3 depletion induced hsa-miR-155-5p expression and hsa-miR-155-5p expression decreased ELK3 expression at the RNA level through a conserved target sequence in its 3′-UTR. We further showed that the activities of hsa-miR-155-5p and ELK3 are functionally linked. Pathway analysis indicates that both factors are implicated in related processes, including cancer and angiogenesis. Hsa-miR-155-5p expression and ELK3 depletion have similar effects on expression of known ELK3 target genes, and on in-vitro angiogenesis and wound closure. Bioinformatic analysis of cancer RNA-seq data shows that hsa-miR-155-5p and ELK3 expression are significantly anti-correlated, as would be expected from hsa-miR-155-5p targeting ELK3 RNA. Finally, hypoxia (0% oxygen) down-regulates ELK3 mRNA in a microRNA and hsa-miR-155-5p dependent manner. These results tie ELK3 into the hypoxia response pathway through an oncogenic microRNA and into a circuit implicated in the dynamics of the hypoxic response. This crosstalk could be important for the development of new treatments for a range of pathologies.

## Introduction

Reduction in the availability of oxygen has drastic effects on cells and tissues [1]. Depending on the organ and the distance from blood vessels, the concentration of oxygen varies between 2–9%. Hypoxia occurs with increasing severity below 2% oxygen until the absence of oxygen and anoxia [2]. A common feature of tumours is that they are hypoxic, and cancerous cells are specially adapted to a hypoxic environment [3]. The key regulator of hypoxia is HIF1α (hypoxia inducible factor 1 alpha). In normoxia, HIF1α is degraded, whereas in low oxygen conditions HIF1α is stabilized and translocates to the nucleus, where it interacts with its binding partner HIF1β to form a transcription factor that can regulate gene transcription [4]. This stabilization allows for a rapid response to oxygen deprivation. However, other factors are also involved in the hypoxia response, including the ETS family member ELK3 [5], [6].

ELK3 is an ETS transcription factor that links MAPK kinase signalling to transcription. It is a member of a subfamily of Ternary Complex Factors (TCF) factors whose peculiarity is to interact with the serum response factor (SRF) [7]. First identified as a repressor of c-Fos expression [8], it has been shown to have roles in cell migration through the control of PAI-1 [9], angiogenesis through VEGF [10] and vascular integrity through EGR1 [11]. Normally functioning as a repressor of gene expression by way of two repressor domains [12], [13] it can become an activator after being targeted by MAPK following activation of the Ras signaling pathway [8]. A role in gene regulation under hypoxia was first suggested when it was observed that all previously identified ELK3 target genes were also responsive to hypoxia and it was demonstrated that ELK3 protein was degraded in low oxygen conditions [5]. Subsequently, its importance in the hypoxic response was revealed by a micro-array study, which showed that the majority of the genes that required HIF1α for their regulation under hypoxia also required ELK3 [6]. ELK3 and HIF1α have been proposed to be involved in distinct yet intricately linked hypoxia-induced signalling pathways [14], yet the ways they interact to regulate gene expression is still not fully understood.

The discovery of a class of microRNAs that respond to hypoxia [15] demonstrates a new facet not only to the hypoxic response, but also to novel mechanisms for its regulation with important implications for disease, including cancer [16], [17]. Several cases have been documented of microRNAs targeting key components of the hypoxia pathway [18], [19], [20], [21]. HIF1α is normally regulated at the protein level; however it was shown that a microRNA, hsa-miR-155-5p, can target the 3′UTR of the HIF1α mRNA message [20]. Furthermore, this was revealed to be a built-in feedback loop into the hypoxia system, by the discovery of an HRE at the MIR155HG (BIC) gene promoter, which expresses the miR, and the revelation that miR-155 expression can be driven in hypoxia by HIF1α [20]. More recently, hsa-miR-155-5p has been shown to target pVHL [19], suggesting it is part of a complex management system for the control of hypoxic response pathways. Another microRNA component of this pathway is hsa-miR-210-5p, which is directly regulated by HIF1α [22] and is often described as the master hypoxamir [23], [24]. Interestingly, ELK3 has been suggested to be a target for both hsa-miR-155-5p [25], [26] and hsa-miR-210-5p [23]. Confirmation of this finding would cement ELK3 into the hypoxic response and consolidate the importance of ELK3 in regulation of hypoxia.

In this study, we demonstrate a feedback mechanism involving hsa-miR-155-5p that can regulate the expression of both ELK3 and HIF1α under hypoxia. Our study integrates ELK3 deeper into our understanding of the cellular response to oxygen and underlines its importance in cellular processes and cancer.

## Materials and Methods

### Cell culture and hypoxic treatment

Mouse skin endothelial (SEND) cells (generous gift from Kari Alitalo) were grown in minimal essential medium (MEM), 10% Fetal Calf Serum (FCS) and 40 µg/ml Gentamicin. Human Umbilical Vein Endothelial Cells (HUVEC; ATCC, Cat. CRL1730) were grown in Dulbecco’s Modified Eagle Medium/Hams F-12- Kaighn’s (DMEM/F12K (1:1) supplemented with 100 µg/ml Heparin, 50 µg/ml Endothelial Cell Growth Supplement, 2 mM Glutamine) 10% Fetal Calf Serum (FCS) and 40 µg/ml Gentamycin. The normoxic conditions were: 20% O_2_, 5% CO_2_ and 37°C in a ThermoForma incubator. The hypoxic conditions (0% oxygen) were achieved using the Anaerocult A system (Millipore).

### Northern Blotting

SEND cells [27] were incubated in 0% oxygen for the indicated times, total RNA was extracted, run on 1% agarose-formaldehyde denaturing gels, transferred to nitrocellulose membranes and hybridized with full length mouse Elk3 and RPLPO (Ribosomal Protein, Large, P0) ^32^[P] labelled probes and exposed to films or Phosphoimager screens for quantification. Elk3 (band 3) intensities were corrected for variations in the housekeeping gene RPLPO, normalised to time 0 h and plotted.

### RNAi and miR mimic/inhibitors

HUVEC cells were transfected at 70–80% confluence. For RNA interference (RNAi), cells were transfected with siRNAs (final concentration of 25 nM) using Lipofectamine 2000 (Invitrogen) according to the manufacturer’s instructions. The siRNA against ELK3 (5′-CAUGGUAGUCUAGAUUUA-3′) targets the 3′UTR (made to order from Sigma), the control siRNA is non-targeting (**Table S1 in File S1**). MicroRNA mimics and inhibitors for hsa-miR-155-5p, hsa-miR-210 and their controls (**Table S1 in File S1**) were transfected in the same manner to a final concentration of 5 nM and samples collected after 48 h.

### Chromatin Immunoprecipitation (ChIP)

The protocol was adapted from the Upstate ChIP Assay Kit (Cat. 17–295). HUVEC cells (5–60×106) were cross-linked in normal medium containing 1% paraformaldehyde (Electron Microscopy Sciences) for 10 min at 37°C. Crosslinking was stopped by the addition of glycine (0.125 M final). Cells were washed with PBS and recovered in Triton-X100 lysis buffer. Nuclei were pelleted and resuspended in 500 µl SDS Lysis Buffer. Samples were sonicated with a Bioruptor (Dianode) to shear DNA to 250–500 base-pairs (bp). Cell debris was pelleted and the chromatin recovered. A fraction was removed as input. Chromatin for immunoprecipitation (IP) was incubated with primary anti-hElk3 antibody (#2005– generated in-house using a peptide from the C-terminus - HMPVPIPSLDRAASPVLLSSNSQKS) overnight at 4°C with rotation. 25 µl of Protein A Magnetic beads (Millipore) per sample were washed three times with ChIP dilution Buffer and added to the chromatin. After 6 h incubation at 4°C beads were washed two times in the following buffers: low salt, high salt, LiCl and TE. The beads were resuspended in 250 µl Elution Buffe r and shaken for 30 minutes at room temperature. The eluate was separated from the beads and crosslinking reversed by incubation overnight at 65°C with NaCl (200 mM final). Input samples were reverse cross-linked at this stage also. DNA was purified using QIA quick PCR purification kit (Qiagen, 28106) according to the manufacturer’s protocol.

### Sequencing of ChIP products and data analysis

ChIP-seq libraries were prepared using Truseq ChIP-Seq Sample Prep Kit (Illumina, IP-202-1012) following the manufacturer’s protocol with some modifications. Briefly, 10 ng of ChIP enriched DNA or control DNA were end repaired using T4 DNA polymerase, Klenow DNA polymerase and T4 Poly Nucleotide Kinase. A single ‘A’ nucleotide was added to the 3′ ends of the blunt DNA fragments with Klenow (3′ to 5′exo minus). The ends of the DNA fragments were ligated to double stranded adapters using T4 DNA ligase. The ligated products were enriched by PCR (30 sec at 98°C [10 sec at 98°C, 30 sec at 65°C, 30 sec at 72°C] x 14 cycles; 5 min at 72°C), and then size selected and purified using Agencourt AMPure XP beads (#A63881, Beckman). Prior to analyses, DNA libraries were checked for quality and quantified using a 2100 Bioanalyzer (Agilent). The libraries were loaded in the flow cell at 7pM concentration and clusters were generated using the Cbot (Illumina, SY-301-2002) and sequenced on the Illumina Genome Hiseq2500 as single-end 50 base reads following Illumina’s instructions.

Base calls performed using CASAVA version 1.8.2. ChIP-seq reads were aligned to the hg19 genome assembly using the Bowtie aligner (http://bowtie-bio.sourceforge.net/index.shtml, release < = 1.0.0, with principle parameters: -m 1–strata –best). Peaks were called using the Model-based Analysis of ChIP-Seq (MACS 1.4.2) algorithm (http://liulab.dfci.harvard.edu/MACS/). The dataset density profiles were normalised to 10^7^ tags for visualisation purposes by Homer (http://homer.salk.edu/homer/ngs/annotation.html). Peaks were annotated using Homer and GREAT (http://bejerano.stanford.edu/great/public/html/).

### Plasmids

Fragments of the ELK3 5′UTR or 3′UTR were amplified from HUVEC genomic DNA using the oligonucleotidess described in **Table S2 in File S1**. Corresponding fragments were gel purified and digested with SpeI (3′UTR) or HindIII, NcoI (5′UTR) and inserted into the XbaI site downstream of luciferase (3′UTR) or the HindIII, NcoI site upstream of luciferase (5′UTR) of the pGL3 (Promega) vectors as recorded in **Table S3 in File S1**. Vectors were verified by sequencing. Mutations to the seed sequences of the two miR155 sites in the 3′UTR were made using PCR amplification of pGL3 Prom hELK3 3′UTR with the oligonucleotides detailed in **Table S2 in File S1** and Pfu polymerase (Stratagene). Amplification products were digested with DpnI (NEB) for 1.5 h at 37°C, purified and transformed. Resulting plasmids were checked for the correct modification by sequencing. Multimers of the identified miR-155 target sequences were created using specific oligonucleotides (**Table S2 in File S1**) which were treated with T4 DNA Kinase (NEB) for 30 min 37°C, boiled for 10 min and left to cool. 5′ phosphorylated oligonucleotides were ligated, digested with XbaI and SpeI and cloned into the XbaI site of the pGL3-Control vector (**Table S3 in File S1).**

### Luciferase Reporter Assays

HUVEC cells were seeded at 80% confluence in 6-well plates and the next day co-transfected with  100 ng of the appropriate vectors +500 ng pCMV-Lac Z plasmid as an internal control and 5 nM siRNA (siElk3 3′UTR or siRisk) or 20 nM miRNA (mir-155, miR control or miR-155 inhibiter) using Lipofectamine 2000. Cell extracts were prepared after  36 h and luciferase activities were measured with the Promega luciferase assay system (according to the manufacturer’s protocol) and an EG&G Berthold luminometer, and corrected for transfection efficiency by using β-galactosidase activity as an internal control.

### Quantitative Real-Time PCR

Total RNA was prepared using TRIREAGENT (Invitrogen) according to the manufacturer’s instructions. Gene expression was analysed with one step Quantitative RT-PCR performed on a Lightcycler 480 system (Roche) using the SYBR Green I kit (Roche) as per the manufacturer’s instructions. Amplification specificity was verified by melting curve analysis and the data was obtained using absolute quantification/2^nd^ Derivative Max with Lightcycler software. For ChIP, a standard curve was generated using the input sample and PCR duplicates of the IP were compared to this curve in order to obtain results relative to input. For RNA, all samples were tested in a dilution series to ensure that for every sample the PCR reactions were occurring with optimal efficiency. Values were obtained by using the dilution series from one sample to create a standard curve to which the other samples could be measured against. Normalization was performed using the internal control RPLPO.

### Detection of microRNAs

Total RNA (500 µg) prepared using TRIREAGENT was treated with polymerase A (NEB, Cat. No. M0276L) for 15 min at 37°C to add a poly A tail to the ends of the miRNA. Reverse transcription was then performed using a primer (**Table S2 in File S1**) and AMV reverse transcriptase (Roche) according to the manufacturer’s instructions. Real time QPCR was performed using a universal primer (**Table S2 in File S1**) and a primer specific to the miRNA of interest (**Table S2 in File S1**) according to the following protocol: 15 min 95°C; 40 cycles of 15 sec 95°C, 30 sec 55°C, 30 sec 70°C; on a Lightcycler 480 system (Roche) using the SYBR Green I kit (Roche).

### MTT Assay

HUVEC cell viability was tested using a MTT assay kit (CT01-5, Millipore) according to the manufacturer’s instructions. Briefly, cells were seeded into 8 wells of a 96 well plate at ×5×103 cells/well. Each time point used a different plate and 8 wells were completed with media only. After overnight incubation plates were either assayed immediately (0 hour time point) or placed in either normoxic or hypoxic conditions for 24 or  48 hours. MTT solution was added and after  2 hours, during which the MTT was metabolised, isopropanol with 0.04N HCL was added to solubilize the formazan. After 30 minutes incubation the absorbance was measured at a wavelength of  550 nm.

### HUVEC tube formation assays

Transfected HUVEC were seeded at a concentration of 5500 cells in 75 µl of endothelial growth media per well of a 96 well plate on top of gelled reduced growth factor Basement Membrane Extract (BD Bioscience, Cat. No. 356237) and incubated for 6 h at 37°C in 5% CO2. Photographs were taken using an EVOS XL Core Imaging System for transmitted-light microscope using the x 10 objective. Tube formation was evaluated using Adobe Photoshop CS4 Extended-Ruler software (an average of 5 fields were used).

### In-vitro wound-healing (scratch) assays

HUVEC cells were trypsinised  36 h after transfection, counted and placed in 24 well plates at 100% confluence (200 000 cells/well). Cells were left overnight to attach and scratched with a sterile 200 µl pipette tip. Plates were washed twice with PBS to remove detached cells and incubated in the complete growth medium. Photographs were taken immediately (0 h) and at 12 and  24 h, using an EVOS XL Core Imaging System for a transmitted-light microscope equipped with 4x objective and the migration was evaluated using Adobe Photoshop CS4 Extended-Ruler software (1000 µm = 458 pixels).

### Western blots and antibodies

Immunoblotting was performed as described in Serchov *et al* [14]. The antibody used and dilutions were as follows: rabbit anti-human ELK3-2005 (generated in-house using a peptide fragment from the C-terminus - HMPVPIPSLDRAASPVLLSSNSQKS) 1/1000, mouse anti-human TBP 1/1500 (clone 3TF1/3G3– Active Motif), HIF1α 1/1000 (NB100-134, Novus Biologicals). Films were scanned with a Chemi Genius 2 Bioimaging system and quantified with Quantity 1 software (BioRad).

### Statistical Analysis

All data are average ± the standard deviation of the average. Statistics were performed using unpaired Student’s t-Test.

## Results

### Identification of genome wide ELK3-chromatin interactions by ChIP-seq

**Figure 1:**
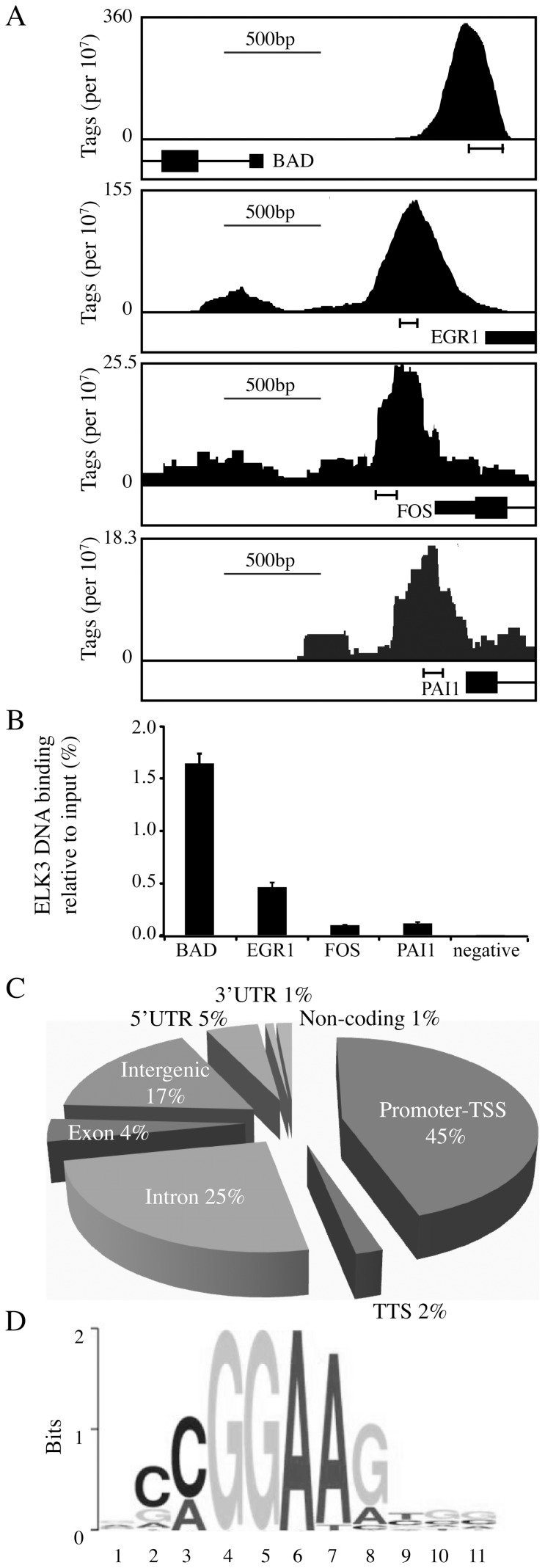
ELK3 DNA binding in HUVEC cells in normoxia. (A) Diagramatical traces of UCSC Genome Browser (http://genome-euro.ucsc.edu/) tracks showing ChIP-seq normalized bedgraphs around the TSS for BAD, EGR1, c-FOS and PAI1. Data is normalised to 10^7^ total tags. Each representation shows 2Kb of sequence data. Precise locations are: BAD - chr11:64,051,548-64,053,547, EGR1 - chr5:137,799,444-137,801,443, FOS - chr14:75,743,992-75,745,991 and PAI1 - chr7:100,768,727-100,770,726. (B) ChIP and QPCR to validate the ChIP-seq. The regions amplified are indicated below the peaks in A. The negative locus is an intergenic location at Chr12: 96463102–96463229 (build: GRCh37/h19) that would not be expected to bind ELK3, and indeed there is no peak in the ChIP-seq data (track not shown). (C) Localisation of ELK3 DNA binding sites as determined by HOMER (http://homer.salk.edu/homer/index.html) annotation of ELK3 peaks (MACS cutoff 10–^10^). (D) Motif analysis by MEME-ChIP (http://meme.nbcr.net/meme/cgi-bin/meme-chip.cgi). The motif shown was found in 707 of 1000 randomly selected peaks and was centrally distributed.

In order to locate ELK3 DNA binding sites on a genome wide scale, we performed a chromatin immunoprecipitation followed by deep sequencing (ChIP-seq) experiment. The antibody was validated by analysing ELK3 binding to the c-FOS promoter (a previously identified target gene [8]) after transfection with either siRNA against ELK3 or a ELK3 over-expression vector. Specificity was shown by decreased binding after treatment with siRNA and increased binding after over-expression (**Figure S1**). Similar results were obtained with two other antibodies against different epitopes of ELK3 (#2620 and Sigma Prestige HPA001600, data not shown). ChIP-seq was performed on HUVEC (human umbilical vein endothelial cells) due to the high endogenous levels of ELK3 and their relevance for the physiological functions of ELK3 in angiogenesis [10], [28], [29]. The ChIP-seq revealed nearly 9 000 peaks when the MACS cut-off score was set to 10–^10^. Selected ChIP-seq peaks were validated in separate experiments by ChIP followed by QPCR (**Figure 1A–B**). We detected ELK3 DNA binding at the locations identified in the ChIP-seq, at levels significantly above the negative control. The 9 000 selected peaks were analysed to determine their location relative to genomic features (HOMER), the presence of centrally located motifs (MEME) and the functions of the closest associated genes. The largest proportion (45%) of the peaks was found to be located between 1 000 bp upstream to  100 bp downstream of transcriptional start sites (**Figure 1C**). The summits of the peaks predominantly contain a centrally located CCGGAAG sequence (**Figure 1D**), which closely matches the ETS-motif, and in particular the sequences recognized by the other TCF family members, ELK1 and ELK4 [30], [31], [32]. ELK3-DNA binding sites are preferentially located near genes involved in “pathways in cancer” (KEGG; Kyoto encyclopedia of genes and genomes) and “angiogenesis” (PANTHER; protein analysis through evolutionary relationships) (**Tables 1** **and** **2**).

**Table 1:** Elk3/hsa-miR-155-5p KEGG pathway correlation.

Pathway Name – Elk3	P-Value	Pathway Name – hsa-miR-155-5p	P-Value
**Pathways in cancer**	**2.60E-06**	Colorectal cancer	2.38E-07
Axon guidance	2.56E-05	**Pathways in cancer**	**1.27E-06**
Endocytosis	4.40E-05	HTLV-I infection	3.18E-06
Insulin signaling pathway	9.46E-05	Renal cell carcinoma	4.11E-06
**Neurotrophin signaling pathway**	**1.12E-04**	T cell receptor signaling pathway	6.55E-06
Fc gamma R-mediated phagocytosis	2.43E-04	B cell receptor signaling pathway	1.34E-05
**Chronic myeloid leukemia**	**3.49E-04**	**Neurotrophin signaling pathway**	**1.53E-05**
**Cell cycle**	**6.94E-04**	mTOR signaling pathway	1.68E-05
Spliceosome	1.02E-03	Endometrial cancer	5.35E-05
Small cell lung cancer	1.34E-03	**MAPK signaling pathway**	**1.24E-04**
**Ubiquitin mediated proteolysis**	**1.47E-03**	Hippo signaling pathway	1.76E-04
Mismatch repair	3.76E-03	Osteoclast differentiation	2.28E-04
Oocyte meiosis	4.35E-03	Pancreatic cancer	2.48E-04
**MAPK signaling pathway**	**5.34E-03**	**Ubiquitin mediated proteolysis**	**1.23E-03**
**Prostate cancer**	**5.54E-03**	**Prostate cancer**	**1.52E-03**
p53 signaling pathway	6.62E-03	Hepatitis B	2.00E-03
Purine metabolism	8.69E-03	Melanoma	2.27E-03
Adherens junction	9.03E-03	**Cell cycle**	**2.54E-03**
Lysosome	1.37E-02	**Chronic myeloid leukemia**	**2.62E-03**
Nucleotide excision repair	1.72E-02	Hedgehog signaling pathway	2.75E-03

Bold highlight = similar pathways, hsa-miR-155-5p information obtained from http://starbase.sysu.edu.cn, Elk3 data obtained by submission to DAVID (http://david.abcc.ncifcrf.gov/home.jsp) of the nearest ENSEMBL gene name to a ChIP-seq peak for the total peak list (MACS-10-05).

**Table 2:** Elk3/hsa-miR-155-5p PANTHER pathway correlation.

Pathway Name – Elk3	P-Value	Pathway Name – hsa-miR-155-5p	P-Value
**Angiogenesis**	**4.42E-04**	Ras Pathway	4.25E-05
VEGF signaling pathway	1.98E-03	Hedgehog signaling pathway	3.16E-04
**PDGF signaling pathway**	**2.33E-03**	**Angiogenesis**	**5.64E-04**
Ubiquitin proteasome pathway	8.70E-03	Interleukin signaling pathway	1.62E-03
Axon guidance	3.17E-02	PI3 kinase pathway	1.91E-03
EGF receptor signaling pathway	3.57E-02	Insulin/IGF pathway	3.38E-03
FAS signaling pathway	6.11E-02	TGF-beta signaling pathway	7.33E-03
Huntington disease	7.83E-02	**PDGF signaling pathway**	**9.47E-03**
Parkinson disease	7.87E-02	Hypoxia response via HIF activation	1.14E-02
Cytoskeletal regulation by Rho	8.18E-02	–	–
Notch signaling pathway	8.41E-02	–	–
Endothelin signaling pathway	8.83E-02	–	–
Circadian clock system	9.04E-02	–	–

Bold highlight = similar pathways, hsa-miR-155-5p information obtained from http://starbase.sysu.edu.cn, Elk3 data obtained by submission to DAVID (http://david.abcc.ncifcrf.gov/home.jsp) of the nearest ENSEMBL gene name to a ChIP-seq peak for the total peak list (MACS-10-05).

### An ELK3/hsa-miR-155-5p regulatory loop

**Figure 2:**
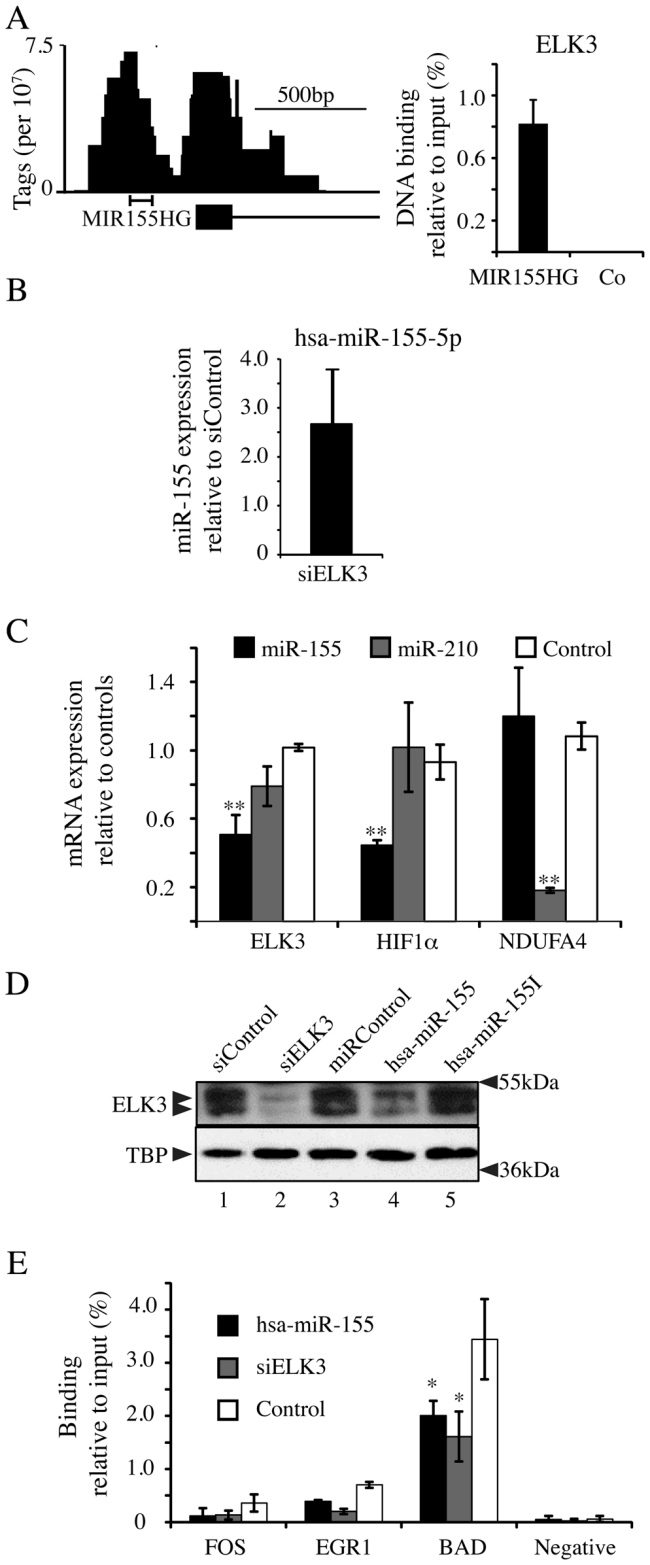
ELK3 feedback loop with hsa-miR-155-5p. (A) ELK3 DNA binding to the BIC (MIR155HG) gene promoter as shown in the ChIP-seq normalised bedgraph and by ChIP-QPCR in comparison to the negative locus at Chr12: 96463102–96463229 (Three separate experiments, error bars represent the S.D. between experiments). (B) miR-155 expression in cells treated with siRNA against ELK3 (Four transfection experiments, error bars represent the S.D.). (C) HUVEC cells treated with miR-155, miR-210 or control miR were analysed for mRNA expression of ELK3, HIF1α and NDUFA4 by RT-QCPR (Three separate transfection experiments, error bars represent S.D., **p<0.01, *p<0.05). (D) Protein expression of ELK3 after treatment with siRNA targeting ELK3, control siRNA, miR-155 mimic, control miRNA or miR-155 inhibitor as described in **Table S1 in File S1**. The loading control was TBP. Quantification of the films from two independent experiments showed that the levels of Elk3 that remained were 40% for siElk3 compared to control, 70% for hsa-miR-155-5p compared to miR Control, and 130% for hsa-miR-155I compared to miRControl. The p values were 0.007, 0.04 and 0.02, respectively, using a Student t-test. (E) ChIP using anti-ELK3 antibody on cells treated with siELK3, miR-155 mimic or control. ELK3 DNA binding was measured at FOS, EGR1, BAD and the negative locus described in Figure 1. Percentage DNA binding compared to input was measured by QPCR (Two separate transfection experiments, error bars represent the standard deviation).

Some of the ELK3-peaks were found to be associated with genes that produce microRNAs (miRs, **Table S4 in File S1**). Since ELK3 regulates gene expression in response to hypoxia [5], we focused on miRs that are regulated by hypoxia [hypoxamirs; **Table S4 in File S1**; [15], [16]]. We selected for further study the MIR155HG (BIC) gene, which produces a hypoxamir that is involved in processes similar to ELK3, including cancer and angiogenesis [**Tables 1** **and** **2**; [19], [33], [34]]. We initially showed that ELK3 negatively regulates MIR-155HG expression. ELK3 was found to bind to the MIR155HG gene promoter in independent ChIP experiments (**Figure 2A** **).** Furthermore, down-regulation of ELK3 with a specific siRNA increased hsa-miR-155-5p expression. (**Figure 2B**). Interestingly, we predicted that hsa-miR-155-5p could also regulate ELK3, since bioinformatics analysis using both miRBase Targets (http://www.ebi.ac.uk/enright-srv/microcosm/htdocs/targets/v5/ *)* and TarBase (http://www.microrna.gr/tarbase, [35]) identified several potential target sites (see below). To test this hypothesis, we compared the ability of hsa-miR-155-5p to down regulate ELK3 mRNA expression with hsa-miR-210-5p, for which ELK3 is a validated target in HUVEC [23]. miRNA mimics were transfected into HUVEC cells and after  48 hours RNA was extracted and the level of ELK3, HIF1α and NDUFA4 mRNA was measured. As expected, hsa-miR-155-5p specifically down-regulated HIF1α, whereas hsa-miR-210-5p specifically down-regulated NDUFA4 [**Figure 2C** [20], [36]]. ELK3 mRNA was down regulated to a greater extent by hsa-miR-155-5p than hsa-miR-210-5p. We tested whether ELK3 expression at the protein level was also decreased by hsa-miR-155-5p. HUVEC cells were transfected with hsa-miR-155-5p mimic, inhibitor, siRNA against ELK3 or the corresponding controls, and endogenous ELK3 protein levels were measured by western blotting. TBP was used as a loading control. Hsa-miR-155-5p significantly decreased ELK3 protein levels, whereas the inhibitor increased ELK3 levels to a small extent (**Figure 2D**). We also tested whether hsa-miR-155-5p reduces binding of ELK3 to target gene promoters. Expression of the hsa-miR-155-5p mimic and siRNA against ELK3 reduced ELK3 binding to the FOS, EGR1 and BAD gene promoters in ChIP experiments (**Figure 2E**). These results show that ELK3 regulates the MIR-155HG (BIC) gene, and that the gene product, hsa-miR-155-5p, can regulate ELK3 expression, forming a double-negative feedback loop.

### Presence of a functional hsa-miR-155-5p target site in the 3′UTR of ELK3 mRNA

**Figure 3:**
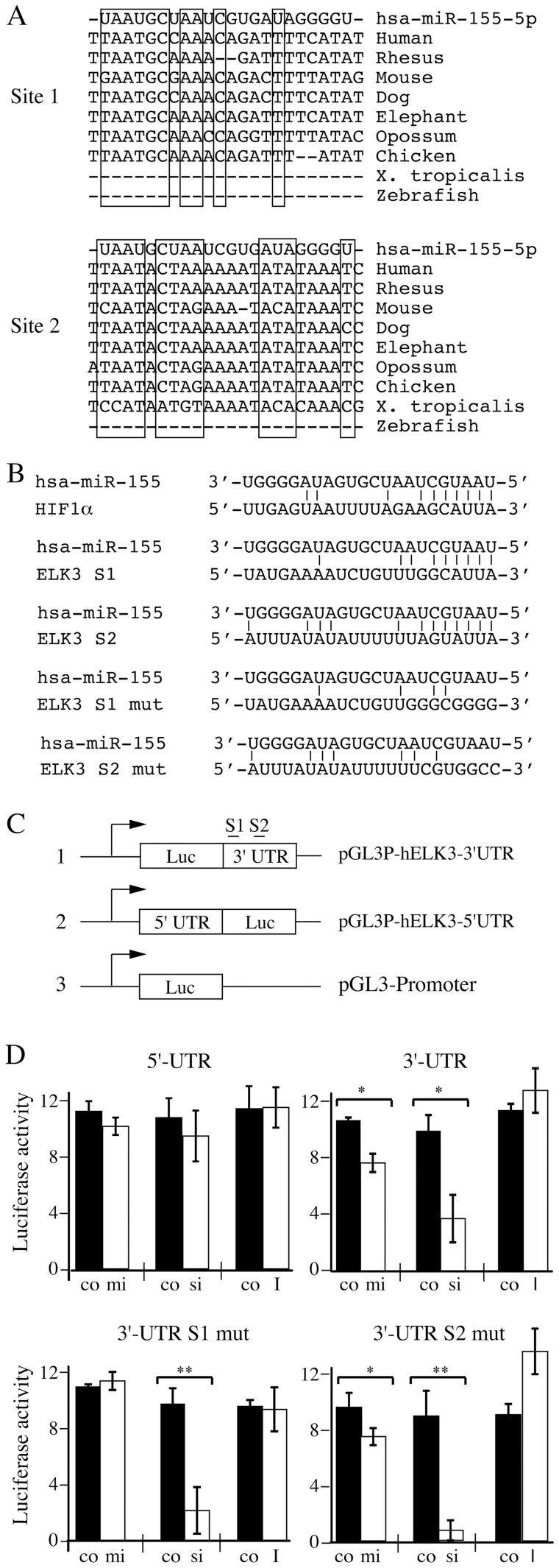
Identification of hsa-miR-155-5p target sites on the ELK3 3′-UTR. (A) Homology between hsa-miR-155-5p and two sequences (S1 & S2) in the human ELK3 gene and conservation in other genera. The RNA sequence of hsa-miR-155-5p and the non-coding sequences of the genes are shown, and the boxes indicate blocks of conservation. The UCSC genome browser was used to locate the predicted target sequences. (B) Complementarity between hsa-miR-155-5p and the HIF1α site identified by Bruning *et al*, [20], S1 & S2 in the ELK3 3′UTR, and the mutants S1 mut and S2 mut. Potential base-pairs are indicated. (C) Schematic representations of the luciferase reporter constructs with the locations of the target sites S1 & S2 in the ELK3 3′UTR. (D) Luciferase expression of the different constructs (5′UTR = pGL3P-hELK3-5’UTR; 3′-UTR = pGL3P-hELK3-3’UTR, 3′ UTR S1 mut = pGL3P-hELK3-3’UTR-S1mut; 3′-UTR S2 mut = pGL3P-hELK3-3’UTR-S2mut) after cotransfection with hsa-miR-155-5p mimic (mi), siELK3 (si), miR155 inhibitor (I) (white bars) or the corresponding control mimics, siRNAs and inhibitors (co, black bars). The experiment was done 3 times (**p<0.01, *p<0.05). Error bars represent the standard deviation in one representative experiment.

Computational analysis identified two potential hsa-miR-155-5p target sites in the 3′UTR of ELK3 (S1 and S2; **Figure 3A–B** **,** the Elk3 gene non-coding strands are shown to facilitate comparison with hsa-miR-155-5p). We found that the sites are conserved from humans to chicken (and Xenopus for Site 2), suggesting that they are functional. In order to test whether they mediate the effects of hsa-miR-155-5p, reporters were constructed with the 3′UTR downstream of luciferase coding sequences in pGL3-promoter (**Figure 3C** and Materials and Methods). Additional constructs were made in which the seed sequences were mutated, thereby decreasing complementarity to hsa-miR-155-5p **(** **Figure 3B**). As expected, transfection with a siRNA targeting the 3′UTR of ELK3 significantly reduced luciferase expression from vectors containing the 3′UTR, but not the 5′UTR or no UTR (**Figure 3D**, data not shown). Transfection of the hsa-miR-155-5p mimic (mi) reduced luciferase expression relative to the control (co) for the construct containing the 3′UTR but not the empty vector or the 5′UTR construct. The effect on the 3′UTR construct was lost when the first hsa-miR-155-5p target site was mutated but not the second. Transfection with the hsa-miR-155-5p inhibitor (I) had no significant effect on luciferase expression from the 3′UTR and 5′UTR construct and the vector containing a mutation of the first hsa-miR-155-5p target site S1. There was a reproducible increase from the S2 mutant, which was not investigated further. These data suggest that hsa-miR-155-5p can target the 3′UTR of ELK3 and specifically site S1.

**Figure 4:**
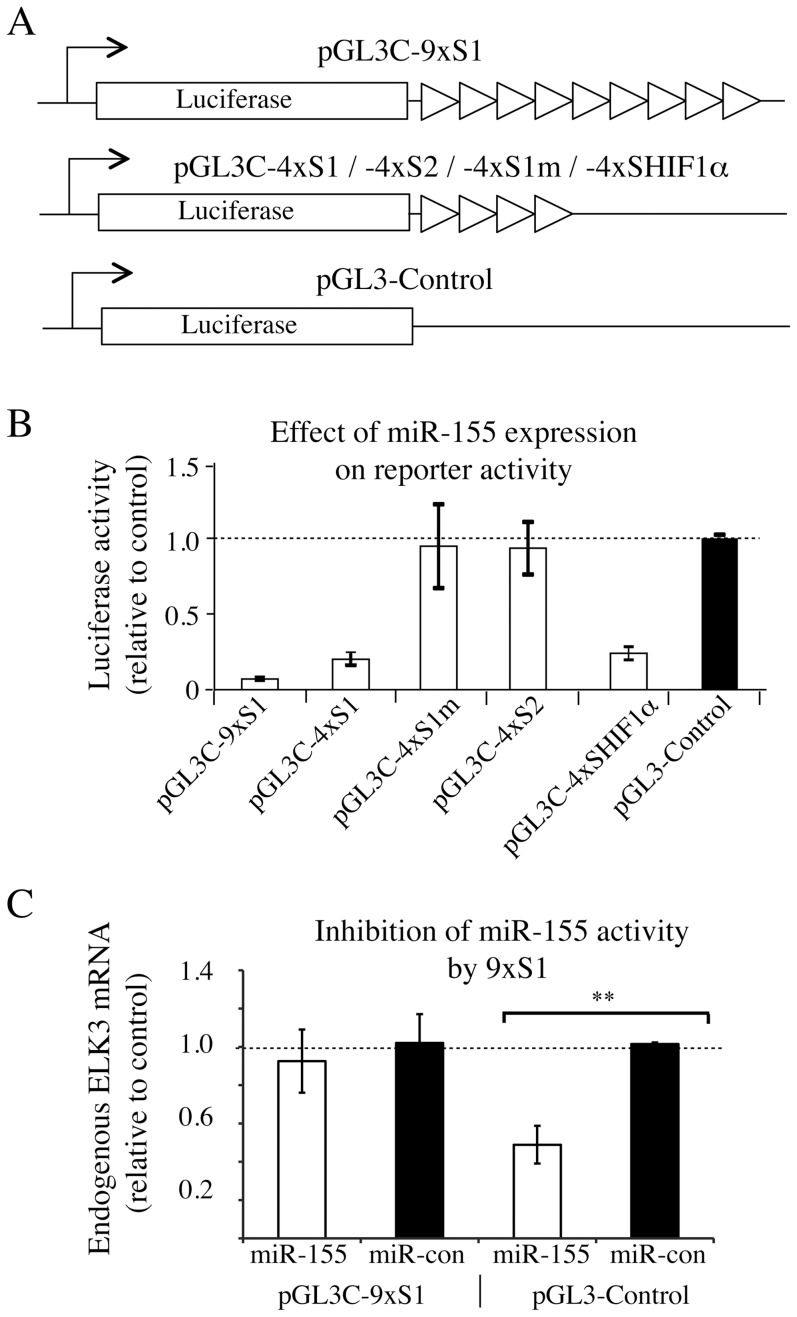
Activities of multimerised ELK3 target sites. (A) Schematic representations of the region of pGL3-Control that contains luciferase coding sequences, the initiation site of the SV40-early promoter and the site of insertion of 4 or 9 copies of S1, S2, S1 mut (S1m) or HIF1α target sequences (Bruning *et al*,) [20]. (B) Effect of miR-155 mimic expression on luciferase expression from the indicated reporters, normalised to the control vector. The error bars represent the S.D. between two separate transfection experiments. (C) Block of miR-155 inhibition of endogenous ELK3 mRNA by overexpression of ELK3 S1 (site 1 miR-155 target sequence). miR-155 mimic or miR control were cotransfected with either the empty control vector or pGL3C-9xS1. Two independent transfection experiments, each analysed twice by qPCR, as described in the Methods section. **p<0.01, error bars represent S.D.).

To study whether the target sites were sufficient, we created luciferase reporter vectors containing multimers of the sites (**Figure 4A**). Expression of the hsa-miR-155-5p mimic decreased luciferase expression from constructs containing 4 copies of S1 or the target sequence of HIF1α, but had no effect when S1 was mutated or when S2 was present. Significantly, a construct containing 9 copies of S1 showed increased sensitivity to the mimic. We also tested whether expression of the Site 1 multimer from the luciferase construct could affect endogenous ELK3 mRNA. Expression of the multimer blocked the ability of transfected hsa-miR-155-5p mimic to decrease endogenous ELK3 mRNA levels, whereas the empty vector did not have an effect (**Figure 4D**). These data suggest that the first hsa-miR-155-5p target site, S1, is the functional site, although we cannot exclude that site 2 may also be functional under certain conditions.

### Functional significance of ELK3 regulation by hsa-miR-155-5p

**Figure 5:**
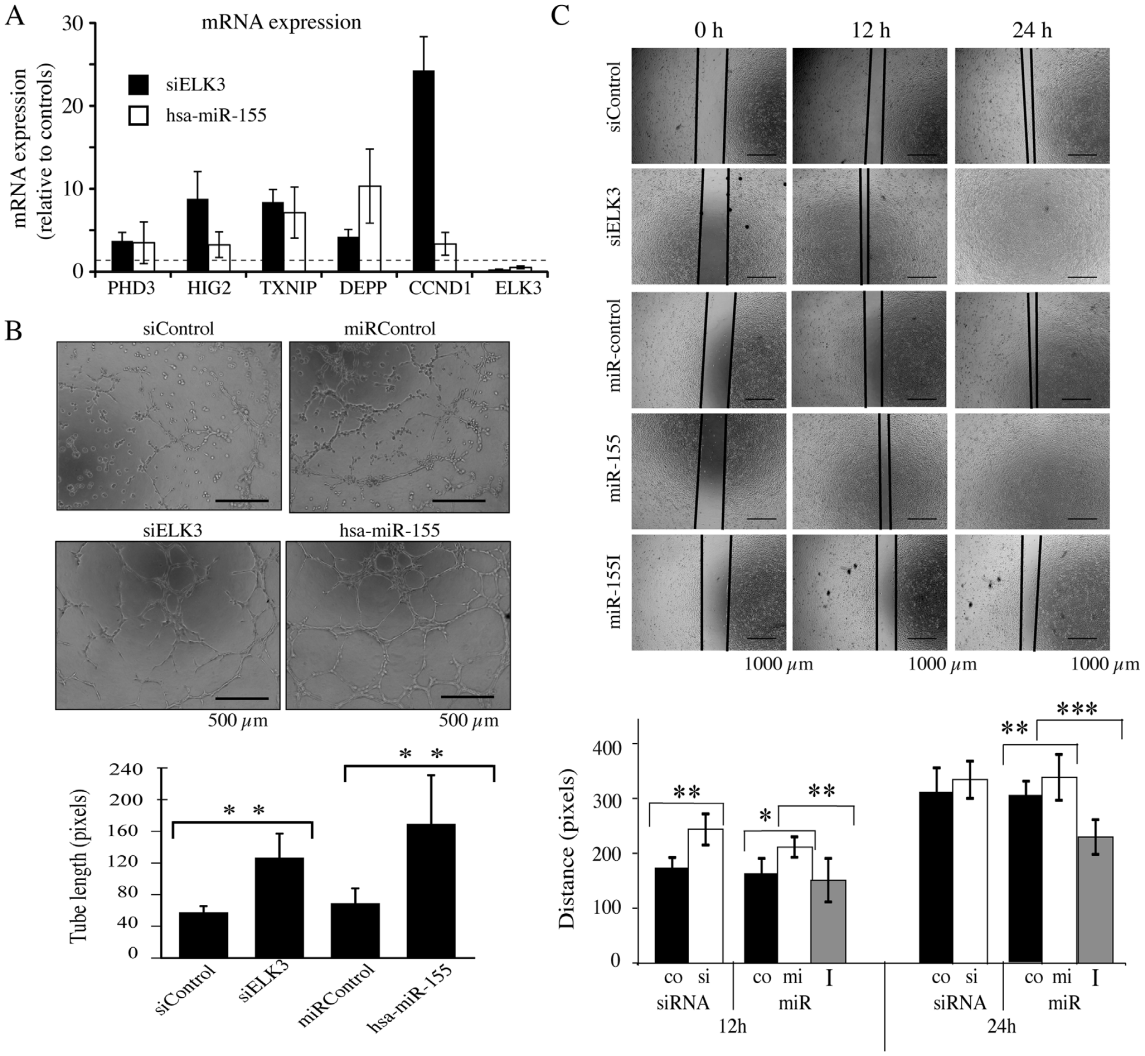
Similar effects of miR-155 mimic expression and ELK3 RNA down-regulation on gene expression, tube formation and migration. (A) mRNA levels of ELK3 target genes, measured by RT-QPCR, 24 h after treatment with either siELK3 or miR-155 mimic, normalised to the corresponding controls (Four separate transfections were analysed for miR-155 and two for siELK3, error bars represent the S.D. between experiments.). (B) Tube formation of HUVEC cells after expression of miR-155 mimics or siELK3 in comparison to the controls (representative pictures, the experiment was repeated 5 times, **p<0.001). The bars represent 500 µm. (C) Cell migration post-transfection with miR-155, siELK3, miR-155 inhibitor or the controls at times 0 h, 12 h and 24 h from the scratch. (Measurements are means of 3 fields per wound. Statistical significance was determined by student *t-*test, the experiment was repeated 3 times, *p<0.01, **p<0.001, ***p<0.0001 error bars represent S.D). The bars represent 1000 µm.

ELK3 and hsa-miR-155-5p are predicted to be involved in related processes, including pathways in cancer (KEGG pathway) and angiogenesis (PANTHER pathway; **Tables 1** **and** **2**). These similarities were investigated by comparing their effects on gene expression, involvement in several cellular processes (tube formation, wound closure), expression patterns in cancer and response to hypoxia. We found that down-regulation of ELK3 by siRNA and expression of hsa-miR-155-5p had similar up-regulatory effects on expression of known ELK3 target genes [PHD3, HIG2, TXNIP, DEPP and CCND1, **Figure 5A** **;** [6]]. HUVEC cells form tubes when grown on matrigel, in a process thought to resemble angiogenesis *in vivo*. Down-regulation of ELK3 by siRNA and overexpression of hsa-miR-155-5p had similar stimulatory effects on tube length (**Figure 5B**) and the % of cells involved in cord formation (data not shown). Wound closure after scratching confluent HUVEC cell cultures was similarly stimulated by down-regulation of ELK3 with siRNA and expression of hsa-miR-155-5p (**Figure 5C** **).** Inhibition of endogenous hsa-miR-155-5p with an inhibitor decreased the rate of wound closure. Overall, these results show that down-regulation of ELK3 can have similar effects as increased expression of hsa-miR-155-5p, as might be expected from direct targeting of ELK3 by this miRNA.

**Figure 6:**
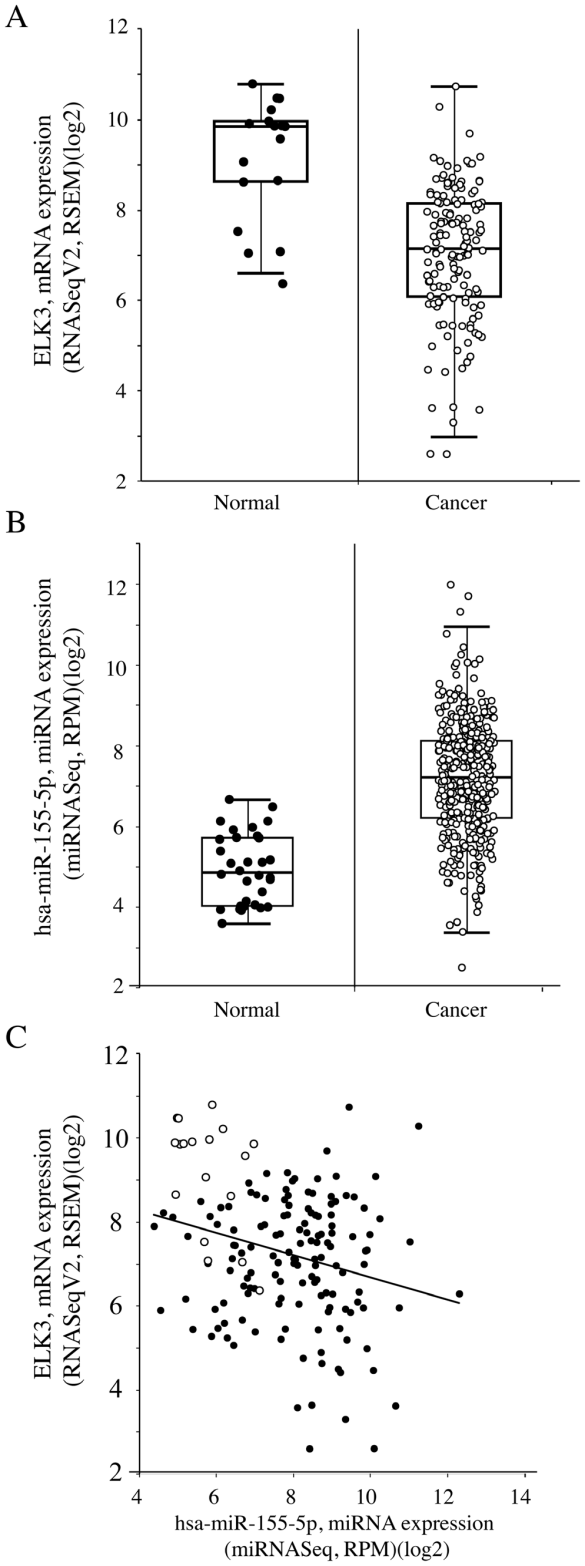
Comparison of ELK3 and miR-155 expression in uterine corpus endometrial carcinoma (UCEC). Corresponding normal samples Expression levels were compared using the TCGA dataset and algorithms available at http://starbase.sysu.edu.cn. (A) Decreased levels of ELK3 in cancer (145) compared to normal (18) samples(fold change = 0.225, student t-test P-Value = 2.80028e-08). (B) Increased levels of hsa-miR-155-5p in cancer (387) compared to normal (32) samples (fold change = 4.669, student t-test P-Value = 5.40827e-17). (C) Anti-correlation between ELK3 and hsa-miR-155-5p levels 1n cancer (Pearson correlation with r = 0.255, P-Value = 0.001).

The expression levels of ELK3 and hsa-miR-155-5p should anti-correlate in conditions where they interact. Using publically available large datasets [Starbase [25]], we found significant anti-correlations in breast cancer (BRCA), head and neck squamous cell carcinoma (HNSCC) and uterine corpus endometrial carcinoma (UCEC) (**Table 3**). In UCEC cancer samples compared to normal, there are lower levels of ELK3 (**Figure 6A** **)** and higher levels of hsa-miR-155-5p (**Figure 6B**). There is an anti-correlation between ELK3 and hsa-miR-155-5p in cancer (**Figure 6C** **).** These results raise the possibility that interactions between ELK3 and hsa-miR-155-5p may be functionally relevant in these cancers.

**Table 3:** Elk3:hsa-miR-155-5p Pearson correlations in cancer.

Cancer Type	Sample Number	R	p-value
Breast cancer (BRCA)	748	−0.12283	0.000761416
Head and neck squamous cell carcinoma (HNSC)	428	−0.09944	0.0397535
Uterine corpus endometrial carcinoma (UCEC)	161	−0.25464	0.0011152

**Figure 7:**
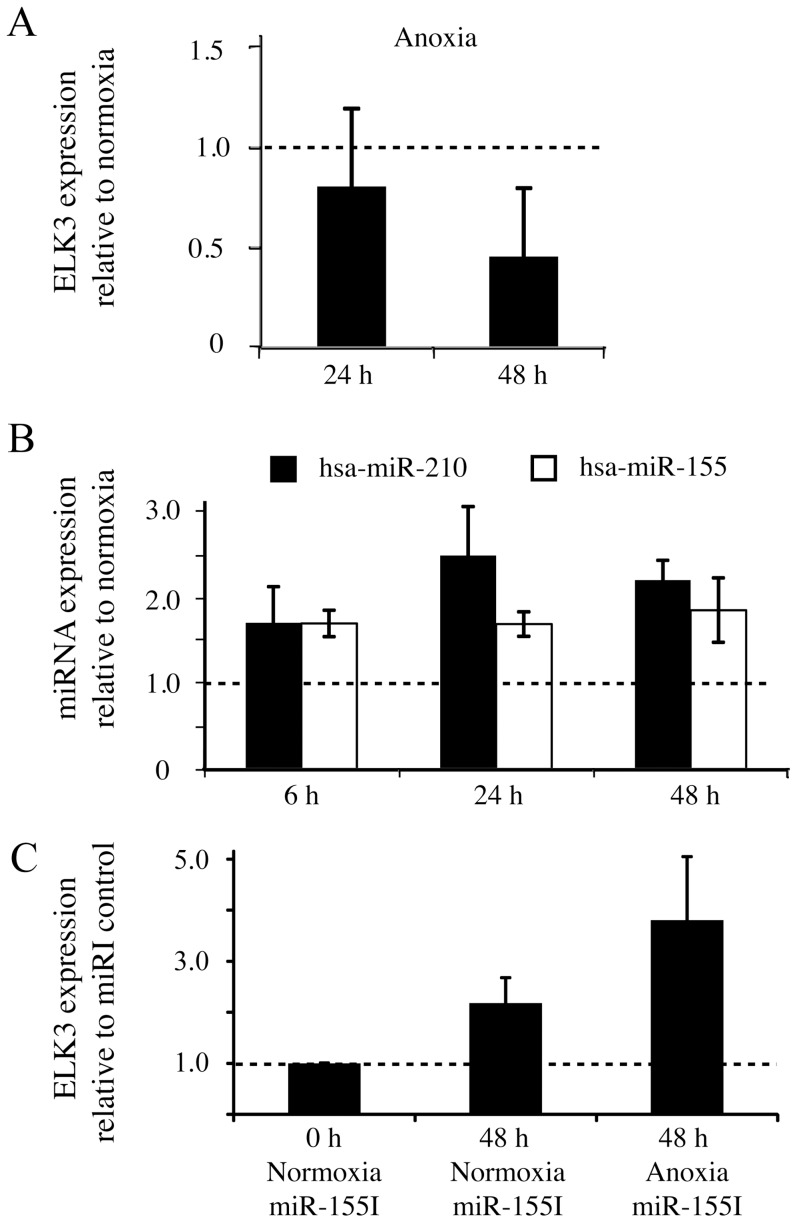
ELK3 mRNA in HUVEC cells is downregulated in anoxia by a mechanism that involves hsa-miR-155-5p. (A) ELK3 mRNA expression in HUVEC at 0% oxygen normalised to  0 h in normoxia (Four separate experiments, error bars represent S.D.). (B) Induction of hypoxamir hsa-miR210-5p and hsa-miR155-5p expression in 0% oxygen. Three separate experiments, error bars represent the S.D. between experiments. miR expression is normalized to normoxia  0 h. (C) ELK3 mRNA expression under anoxia (0% oxygen) after treatment with the miR-155 inhibitor (miR-155I, see **Table S1 in File S1**) relative to the inhibitor control. Three separate experiments, error bars represent the S.D.

Hsa-miR-155-5p is a hypoxamir [19], [20], raising the possibility that it could regulate ELK3 in hypoxia. Although ELK3 mRNA is stable in 1% oxygen hypoxia for  24 h in SEND endothelial cells [5], [14], we have evidence for specific degradation of ELK3 mRNA in more extreme conditions (anoxia, 0% oxygen) in these cells (**Figure S2A–B**). Therefore, we examined the effects of very low oxygen in HUVEC. Anoxia induces rapid stabilization of HIF1α and the up-regulation of its target gene VEGF (**Figure S2C–D**), a marked decrease in ELK3 mRNA expression over  48 hours (**Figure 7A**) and up regulation of hsa-miR-155-5p and hsa-miR-210 (**Figure 7B**). The hypoxia conditions do not cause extensive cell death, but rather reversible inhibition of proliferation, as shown by MTT assays of cells exposed to normoxia and hypoxia for 24 and  48 hours, and recovery for  24 hours in normoxia following  48 hours in hypoxia (**Figure S2E**). In order to determine whether a microRNA was involved in regulation of ELK3 mRNA in anoxia, we disrupted the microRNA processing machinery by down-regulating the essential factor Dicer [37]. Dicer knockdown with a specific siRNA (**Figure 7C** **)** decreased the levels of mature hsa-miR-155-5p and hsa-miR-210-5p and increased ELK3 mRNA after  48 hours of anoxia. In order to determine whether hsa-miR-155-5p was involved, we blocked endogenous hsa-miR-155-5p with a specific inhibitor. Inhibition of hsa-miR-155-5p resulted in increased Elk3 mRNA levels that were greater in anoxia than in normoxia (**Figure 7D**). These results show that there is a miRNA dependent decrease in ELK3 mRNA in anoxia that involves hsa-miR-155-5p.

## Discussion

In this study, we have discovered a new facet to the traditional hypoxia pathway and one which links ELK3 and HIF1α even more closely in terms of their regulation under hypoxic conditions. Furthermore we have identified a novel target for hsa-miR-155-5p, an oncomir, which has shown potential for being a prognostic factor for some cancers [38]. Specifically, we have shown that ELK3 and hsa-miR-155-5p are part of a feedback loop, which may be important for a complete understanding of the cellular response to hypoxia and importantly, comprehension of the hypoxic tumour microenvironment. This feedback system could be important in the formation and/or maintenance of the tumour vasculature and therefore it may lead to better therapies targeting the vaculature.

We identified two conserved hsa-miR-155-5p target sites in the 3′UTR of ELK3, however we were only able to show one of these as being functional. The reasons for this are unclear, as both sequences are very similar; only a better understanding of miRNA targeting will provide an explanation. It is possible that the second site may be active in a different context, or that additional co-factors or 3D structures may be required that are not reproduced in our assays.

ChIP-seq has been performed on the ternary complex factors [30], [31], [32], [39]. Ours is the first genome-wide analysis of ELK3-DNA interactions that has been used to uncover new properties of ELK3. There is interplay between the TCFs at their binding sites [40], and more comprehensive analysis is required to resolve the relationship between these factors. The identified DNA binding motif for ELK3 is similar, but not identical, to those of ELK1 and ELK4. Whilst the central CCGGAA is conserved between the three factors, the importance of the individual base pairs and the surrounding sequences of the motifs are subtly different. It is known that the different TCF family members can occupy the same sites, although whether this is due to redundancy between the factors or if they perform different roles at the same promoters remains to be determined. Given the high level of redundancy between the TCF family members, an important caveat is that some of the sites identified may only be conditional targets of ELK3. The slight differences in the DNA binding sites of the three factors could mean that some sites favour one factor over the other. The functions of the three factors could also differ in the way they are targeted by miRNAs. Interestingly, only ELK3 mRNA has hsa-miR-155-5p, as determined by database searches of miRbase Targets and Targetscan. Completing this form of analysis for the TCF family will hopefully help to reveal the interplay between and specificities of these three factors.

Whilst we have described the targeting of ELK3 by hsa-miR-155-5p, there are many other microRNAs that may be able to regulate ELK3 expression, and some of these may even be hypoxia regulated. For example hsa-miR23A was identified by Targetscan as having the potential to target ELK3, is reported as being hypoxia regulated [15] and is perhaps directly regulated by ELK3 (**Table S4 in File S1**). ELK3 has been identified experimentally as a target of another hypoxamir, hsa-miR-210-5p, although there was only a small effect on ELK3 mRNA and no changes at the protein level [23]. We found at most small changes in ELK3 mRNA expression with these miRNAs, which were however not statistically significant (data not shown). It could be that our methods are not sensitive enough to accurately detect these changes. It is also possible that under different conditions the effect of on ELK3 could be more pronounced, as at-yet little is known about how microRNAs are selective between all their potential targets. These unanswered questions leave significant scope for more in-depth analysis to determine all the potential microRNAs or hypoxamirs that may regulate ELK3.

Downregulation of Elk3 by siRNA and hsa-miR-155-5p did not always results in identical effects on the expression of Elk3 target genes (**Figure 5A**). There could be a number of explanations for this, and indeed treatment with siELK3 and miR-155 would not necessarily be expected to have the same effect. It is known that hsa-miR-155-5p has many other targets, such as HIF1α, which could explain some of the differences. Additionally it is possible that hsa-miR-155-5p may target one of the genes directly. Cyclin D1 has recently been identified as a target for miR-155 [41]. However, we predicted that the two treatments should be broadly similar, and indeed that is what we observe.

As reported in Bruning *et al* [20], HIF1α can both regulate the expression of hsa-miR-155-5p in hypoxia by binding to an HRE site in the promoter of the MIR155HG (BIC) gene under hypoxic conditions and in turn be regulated by hsa-miR-155-5p through a specific target site in the HIF1α 3′UTR. Here we report a related situation for ELK3 with a specific target sequence for hsa-miR-155-5p in the 3′UTR that mediates ELK3 mRNA down regulation when hsa-miR-155-5p expression increases. We also identified a role for ELK3 in regulating the expression of this microRNA, although conversely to HIF1α, since ELK3 acts as a repressor of miR expression under normal conditions. We have previously reported on the similarity in the control of gene expression between ELK3 and HIF1α with both proteins controlling the expression of many of the same genes under hypoxia [6]. Here, we identified another link between the two factors and suggest a common mechanism for regulation through hsa-miR-155-5p. These data tie ELK3 ever closer into the HIF1α regulatory pathway.

Whilst hsa-miR-155-5p is a known hypoxamir, it is known that other factors can induce its expression. Induction of hsa-miR-155-5p by TNFα through NFκβ in 3T3-L1 preadipocytes results in the inhibition of adipogenesis [42]. It could be that induction of hsa-miR-155-5p under different circumstances could also imply a downstream role for ELK3 in those pathways. The nature of the ELK3-hsa-miR-155-5p relationship under different conditions could be as important as the one we have described here under hypoxia.

Hsa-miR-155-5p has been reported to affect various physiological and pathological processes, such as hematopoietic lineage differentiation, immunity, inflammation, viral infections, cancer, cardiovascular disease and Down syndrome. Hsa-miR-155-5p can act as an oncogene, but there are also reports of it having tumour suppressor functions [for reviews see [43], [44], [45], [46]]. The interactions between hsa-miR-155-5p and ELK3 may be expected to be relevant in some of these. From the current work and previous studies, directions to follow would be several types of cancer (Ovarian, Breast and HNSCC), angiogenesis and possibly hematopoiesis. In previous studies, we have shown that mice lacking Elk3 have altered blood cell content when exposed to a hypoxia mimic [5].

**Figure 8:**
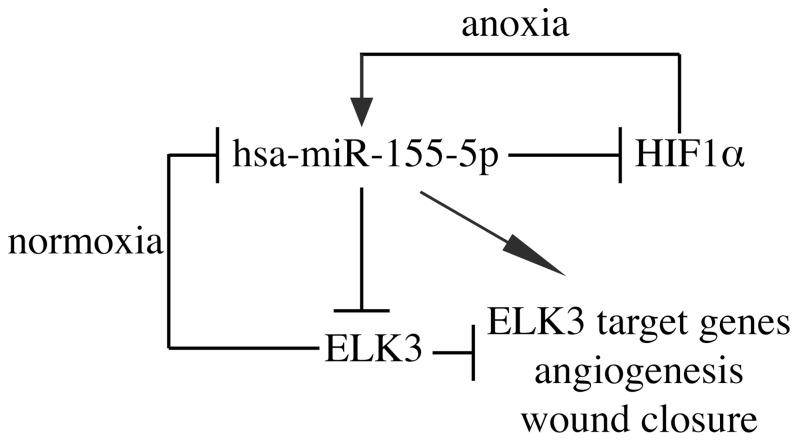
Model of the anoxia-ELK3-miR155 regulatory circuit. ELK3 blocks hsa-miR-155-5p expression in normoxia. Under anoxic conditions, hsa-miR-155-5p expression increases due to HIF1α induction, which in turn leads to decreased HIF1α and ELK3 mRNA expression. Increased hsa-miR-155-5p expression and decreased Elk3 expression induce ELK3 target genes, angiogenesis and cell migration.

From our results, we are able to suggest a basic model (**Figure 8**). Under normoxic conditions, ELK3 represses hsa-miR-155-5p expression. However, under hypoxia, HIF1α is stabilized and induces hsa-miR-155-5p expression. In turn, hsa-miR-155-5p feeds-back to inhibit HIF1α and ELK3 mRNA expression, and to increase ELK3 target gene expression, and in-vitro angiogenesis and wound closure. A key difference is that for HIF1α the effect of the feedback is negative whilst for ELK3 it is double-negative. Double-negative feedback loops are known to exhibit bistable behaviour and are important in developmental transcriptional regulation [47]. Interpreting the action of this model under different hypoxic conditions will require further work. This model omits some important subtleties. Firstly, we know that the degradation of ELK3 requires the oxygen concentration to be below 1%, whereas the induction of hsa-miR-155-5p in hypoxia can occur at 1% oxygen. Therefore, there is a graded response depending on the severity of hypoxia. ELK3 protein has been shown to be transported from the nucleus and degraded under hypoxia. Presumably degradation of the mRNA under severe hypoxic conditions has implications for the recovery time on the return to normal oxygen conditions.

In conclusion we have identified a novel mechanism for regulation of ELK3 under hypoxic conditions and this mechanism is shared with the hypoxic response factor HIF1α. We have already provided evidence that these two proteins have a close relationship in the regulation of the hypoxic response and here we have identified another strong link. Furthermore, we have discovered anti-correlations between hsa-miR-155-5p and ELK3 in some types of cancer, suggesting that this relationship has an important role in some pathologies, which could potentially be exploited for the development of therapies.

## Supporting Information

File S1 **Contains supporting tables.** Table S1, siRNA/miR mimics/inhibitors. Suppliers and catalogue numbers are given. Table S2, Oligonucleotides. Oligonucleotides were designed using Primer3 (http://bioinfo.ut.ee/primer3-0.4.0/) or integrated design technologies realtime PCR tool and subsequently purchased from Sigma. Table S3, Vectors. The vectors used in this work are listed in the table, and are either based on a commercially available backbone or were described previously [1]. Table 4, Hypoxamirs. Highlighted in blue are hypoxamirs identified by Kulshreshtha et al [2], and highlighted in red are hypoxamirs identified by Shen et al. [3].
(DOCX)

Figure S1 **Validation of the ELK3 antibody for ChIP-seq.** (A) ChIP on HUVEC cells transfected with siRNA against ELK3 (siELK3) or overexpression plasmid (pTL2hELK3) and the corresponding controls (siCont and pTL2). DNA in the immunoprecipitates were analysed by QPCR with primers for a region in the c-FOS promoter and a negative control DNA region expected not to bind ELK3.
(TIF)

Figure S2 **ELK3 mRNA degradation in anoxia (0% oxygen) in SEND cells.** (A) Northern blot of total RNA extracted from SEND cells cultured in 0% oxygen for the designated times and probed for ELK3 and RPLPO. The locations of 18S and 28S ribosomal RNA are indicated. (B) Northern Blot quantification. The major Elk3 band (3, 1,600 bases) was quantified by scanning suitably exposed autoradiograms, correcting for RPLPO, and normalizing to the zero time point. One representative example of four independent experiments is shown. (C) HIF1α induction under 0% oxygen conditions. HUVEC cells were incubated in 0% oxygen for the indicated times and whole cell extracts were analysed by western blotting with antibodies against HIF1α and TBP as a loading control. (D) VEGF mRNA induction under 0% oxygen conditions. HUVEC cells were incubated in 0% oxygen for the indicated times, and extracted RNA was analysed by RT-QPCR for VEGF and RPLPO RNA levels. VEGF RNA levels were corrected for the internal control RPLPO, and normalized to the  0 h time point. (E) HUVEC cells viability during hypoxia in a MTT assay in comparison to media without cells. Measurements were taken at the start of the experiment (lane 1) and after 24 and  48 hours of normoxia (lanes 2 and 3, respectively) or hypoxia (lanes 4 and 5, respsctively). A further point was taken after  24 hours of re-oxygenation (lanes 6). Error bars represent the S.D. within the experiment.
(TIF)
