# Supplementary material for: The Oncogenic MicroRNA Hsa-miR-155-5p Targets the Transcription Factor ELK3 and Links It to the Hypoxia Response
Source: PLoS One. 2014 Nov 17;9(11):e113050. doi: 10.1371/journal.pone.0113050 (PMC4234625; doi:10.1371/journal.pone.0113050)
Supplement: File S1 — Contains supporting tables. Table S1, siRNA/miR mimics/inhibitors. Suppliers and catalogue numbers are given. Table S2, Oligonucleotides. Oligonucleotides were designed using Primer3 (http://bioinfo.ut.ee/primer3-0.4.0/) or integrated design technologies realtime PCR tool and subsequently purchased from Sigma. Table S3, Vectors. The vectors used in this work are listed in the table, and are either based on a commercially available backbone or were described previously [1]. Table 4, Hypoxamirs. Highlighted in blue are hypoxamirs identified by Kulshreshtha et al [2], and highlighted in red are hypoxamirs identified by Shen et al. [3]. (DOCX) [file pone.0113050.s001.docx]

**Supplementary Tables**

**Table S1. siRNA/miR mimics/inhibitors.**

| **Name** | **Supplier** | **Catalogue Number** |
| --- | --- | --- |
| siELK3 3’ UTR | Sigma | N/A |
| siControl (SiGenome RISC-Free Control) | Thermo Scientific | D-001220-01-20 |
| hsa-miR-155-5p mimic | Thermo Scientific | C-300647-05-0005 |
| hsa-miR-210-5p mimic | Thermo Scientific | C-300565-03-0005 |
| hsa-miR-155-5p inhibitor | Thermo Scientific | IH-301071-02-0005 |
| miR Mimic Control | Thermo Scientific | CN-001000-01-05 |
| miR Inhibitor Control | Thermo Scientific | IN-001005-01-05 |

**Table S2. Oligonucleotides.**

| **Gene** | **Sequence** | **Use** |
| --- | --- | --- |
| ELK3 F | 5’-AGTCCACTGCTCTCCAGCAT-3’ | RT-QPCR |
| ELK3 R | 5’-GCCAGACGTCATCAGGATTT-3’ | RT-QPCR |
| RPLPO F | 5’-GAAGGCTGTGGTGCTGATGG-3’ | RT-QPCR |
| RPLPO R | 5’-CCGGATATGAGGCAGCAGTT-3’ | RT-QPCR |
| DICER F | 5’-TGGGTCCTTTCTTTGGACTG-3’ | RT-QPCR |
| DICER R | 5’-CTGGTTTGCAGAGTTGACCA-3’ | RT-QPCR |
| HIF1α F | 5’-CCGCTGGAGACACAATCATATC-3’ | RT-QPCR |
| HIF1α R | 5’-ACTTCCTCAAGTTGCTGGTC-3’ | RT-QPCR |
| NDUFA4 | 5’-AGCTTGATCCCCCTCTTTGT-3’ | RT-QPCR |
| NDUFA4 | 5’-CTGGACGTTCCTTCTTCAGC-3’ | RT-QPCR |
| PHD3 F | 5’-AGATCGTAGGAACCCACACG-3’ | RT-QPCR |
| PHD3 R | 5’-CAGATTTCAGAGCACGGTCA-3’ | RT-QPCR |
| DEPP F | 5’-GACTGTCCCTGCTCATCCAT-3’ | RT-QPCR |
| DEPP R | 5’-GGCTGTGCCAGTCGAGATA-3’ | RT-QPCR |
| HIG2 F | 5’-GTGCTCGCGGCTATAAGG-3’ | RT-QPCR |
| HIG2 R | 5’-AACACATGCTTCATGGCTGA-3’ | RT-QPCR |
| TXNIP F | 5’-TGACATGGATGGCTCTCAAG-3’ | RT-QPCR |
| TXNIP R | 5’-TTCTTCCACATGCTCACTGC-3’ | RT-QPCR |
| CCND1 F | 5’-GATCAAGTGTGACCCGGACT-3’ | RT-QPCR |
| CCND1 R | 5’-TCCTCCTCTTCCTCCTCCTC-3’ | RT-QPCR |
| miR155 | 5’-TTAATGCTAATCGTGATAGGGGT-3’ | miR-RT-QPCR |
|  | 5’- GCTGTCAACGATACGCTACGTAACGGCATGACAGTGTTTTTTTTTTTTTTTTTTTTTTTTVN-3’ | miR reverse transcriptase primer |
|  | 5’-GCTGTCAACGATACGCTACGTAACG-3’ | miR universal primer |
| miR210 | 5’- AGCCCCTGCCCACCGCACACTG -3’ | miR-RT-QPCR |
| RNU44 | 5’-CCTGGATGATGATAAGCAAATG-3’ | miR-RT-QPCR |
| BAD F | 5’-GCCAATTCTCGTACGGTTTC-3’ | ChIP |
| BAD R | 5’-AGGTCCTCAGGCCGCTGAT-3’ | ChIP |
| EGR1 F | 5’-GCCATATAAGGAGCAGGAAGG-3’ | ChIP |
| EGR1 R | 5’-CTTCTTCCCTCCTCCCAGAG-3’ | ChIP |
| FOS F | 5’-GAGCAGTTCCCGTCAATCC-3’ | ChIP |
| FOS R | 5’-CCCCAAGATGAGGGGTTT-3’ | ChIP |
| Negative | 5’-CGGTGGGGCTCTACAGAAAT-3’ | ChIP |
| Negative | 5’-CCTGAAAGTGGCTCCGAGTA-3’ | ChIP |
| miR155F | 5’-TCATTTGAAGGCGTTTCCTT-3’ | ChIP |
| miR155R | 5’-GGGTCATTGCGTGTGTGTAT-3’ | ChIP |
|  | 5’-GGACTAGTGACAGAGCTGCTTCTCCAGTACT-3’ | Cloning 3’UTR of ELK3 |
|  | 5’-GGACTAGTCGATGTTTATTGTCTAAATATGGCTTAG-3’ | Cloning 3’UTR of ELK3 |
|  | 5’-CCCAAGCTTGGGCGGAAAAGCCTGTTTA-3’ | Cloning 5’UTR of ELK3 |
|  | 5’-CATGCCATGGCAGATGTGGGGGAGTCTTC-3’ | Cloning 5’UTR of ELK3 |
|  | 5’-GGACTAGT ACGCTGCCAGTATTTCATCC-3’ | Cloning miR210 CDS seed of ELK3 |
|  | 5’-GGACTAGT ACTGGACTAAGGCTGCTCC-3’ | Cloning miR210 CDS seed of ELK3 |
|  | 5’-ATTCAAGTATATATGAAAATCTGTTGGGCGGGGAGTGAATTTTAATGTTTTTGTTTT-3’ | miR155 site1 mutation |
|  | 5’-AAAACAAAAACATTAAAATTCACTCCCCGCCCAACAGATTTTCATATATACTTGAAT-3’ | miR155 site1 mutation |
|  | 5’-TAAATGAATGATTTATATATTTTTTCGTGGCCAGAGGAAATGTTTGAAAGATGAAAAT-3’ | miR155 site2 mutation |
|  | 5’-ATTTTCATCTTTCAAACATTTCCTCTGGCCACGAAAAAATATATAAATCATTCATTTA-3’ | miR155 site2 mutation |
|  | 5’-CTAGATATATATGAAAATCTGTTTGGCATTAAGTGAAA-3’ | Multimer miR155 site 1 |
|  | 5’-CTAGTTTCACTTAATGCCAAACAGATTTTCATATATAT-3’ | Multimer miR155 site1 |
|  | 5’-CTAGATGAAATTTATATATTTTTTAGTATTAAGAGGAA-3’ | Multimer miR155 site2 |
|  | 5’-CTAGTTCCTCTTAATACTAAAAAATATATAAATTTCAT-3’ | Multimer miR155 site2 |
|  | 5’-CTAGATATATATGAAAATCTGTTGGGCGGGGAGTGAAA-3’ | Multimer site1 mutated |
|  | 5’-CTAGTTTCACTCCCCGCCCAACAGATTTTCATATATAT-3’ | Multimer site1 mutated |
|  | 5’-CTAGAAATAATTGAGTAATTTTAGAAGCATTATTTTAA-3’ | Multimer miR155 site HIF1α |
|  | 5’-CTAGTTAAAATAATGCTTCTAAAATTACTCAATTATTT-3’ | Multimer miR155 site HIF1α |
|  | 5’- GCTGTCAACGATACGCTACGTAACGGCATGACAGTGTTTTTTTTTTTTTTTTTTTTTTTTVN-3’ | miR reverse transcriptase primer |
|  | 5’-GCTGTCAACGATACGCTACGTAACG-3’ | miR universal primer |

**Table S3. Vectors.**

| **Vector Name** | **Backbone** | **Vendor** |
| --- | --- | --- |
| pTL2 | pTL2 |  |
| pTL2hNET | pTL2 |  |
| pGL3 Prom hELK3 3'UTR | pGL3 promoter | Promega |
| pGL3 Prom hELK3 5'UTR | pGL3 promoter | Promega |
| pGL3 Prom Empty | pGL3 promoter | Promega |
| pGL3 control miR155 hELK3 3'UTR 9x sequence 1 | pGL3 control | Promega |
| pGL3 control miR155 hELK3 3'UTR 4x sequence 1 | pGL3 control | Promega |
| pGL3 control miR155 hELK3 3'UTR 4x mutated sequence 1 | pGL3 control | Promega |
| pGL3 control miR155 HIF1α 3'UTR 4x | pGL3 control | Promega |
| pGL3 control Empty | pGL3 control | Promega |

**Table S4. Hypoxamirs.**

| **Name** | **Peak Score** |
| --- | --- |
| MIR663B | 894,95 |
| MIRLET7A1 | 781,33 |
| MIR320A | 634,55 |
| MIR1260B | 611,08 |
| MIR1181 | 519,11 |
| MIR4444-1 | 512,07 |
| MIR3916 | 509,37 |
| MIR1281 | 506,07 |
| MIR1976 | 504,24 |
| MIR5091 | 490,49 |
| MIR126 | 476,63 |
| MIR23A | 471,52 |
| MIR1539 | 451,49 |
| MIR145 | 433,86 |
| MIR3960 | 423,08 |
| MIR3124 | 403,31 |
| MIR4316 | 386,58 |
| MIR4489 | 356,55 |
| MIR4515 | 339,19 |
| MIR4519 | 298,29 |
| MIR3654 | 292,77 |
| MIR4466 | 290,86 |
| MIR935 | 289,04 |
| MIR3654 | 280,87 |
| MIR4313 | 276,6 |
| MIR193A | 274,81 |
| MIR548A1 | 261,17 |
| MIR1205 | 257 |
| MIR1246 | 255,9 |
| MIR196B | 252,84 |
| MIR658 | 246,94 |
| MIR22HG | 245,15 |
| MIR4673 | 242,74 |
| MIR648 | 241,31 |
| MIR612 | 240,65 |
| MIR30D | 240,35 |
| MIR4669 | 234,17 |
| MIR4674 | 232,6 |
| MIR5190 | 226,63 |
| MIR3193 | 224,42 |
| MIR3191 | 221,75 |
| MIR4710 | 218,96 |
| MIR5010 | 216,99 |
| MIR153-1 | 216,1 |
| MIR4266 | 215,11 |
| MIR4669 | 213,21 |
| MIR3922 | 211,35 |
| MIR99B | 207,12 |
| MIR23A | 202,12 |
| MIR4673 | 201,92 |
| MIR1301 | 201,88 |
| MIR4309 | 201,02 |
| MIR935 | 200,13 |
| MIR4266 | 196,24 |
| MIR4265 | 193,66 |
| MIR648 | 191,87 |
| MIR1229 | 191,84 |
| MIR21 | 191,52 |
| MIR3155A | 191,03 |
| MIR3619 | 189,68 |
| MIR4761 | 188,27 |
| MIR3175 | 187,11 |
| MIR3688-2 | 186,23 |
| MIR23A | 184,99 |
| MIR612 | 184,42 |
| MIR3151 | 184,37 |
| MIR4740 | 183,32 |
| MIR4681 | 182,41 |
| MIR145 | 179,08 |
| MIR4669 | 176,46 |
| MIR202 | 176,18 |
| MIR133A2 | 175,27 |
| MIR125B1 | 172,22 |
| MIR4757 | 171,34 |
| MIR326 | 170,95 |
| MIR365B | 168,83 |
| MIR212 | 168,37 |
| MIR4505 | 167,81 |
| MIR4309 | 166,51 |
| MIR4493 | 165,57 |
| MIR4257 | 165,55 |
| MIR1915 | 164,83 |
| MIR4505 | 163,05 |
| MIR4505 | 162,99 |
| MIR3200 | 162,34 |
| MIR5191 | 162,13 |
| MIR5092 | 159,54 |
| MIR4645 | 159,06 |
| MIR4316 | 156,48 |
| MIR3200 | 156,36 |
| MIR3162 | 155,49 |
| MIR4777 | 155,48 |
| MIR3648 | 153,25 |
| MIR4442 | 151,71 |
| MIR4745 | 151,45 |
| MIR3138 | 150,85 |
| MIR153-2 | 149,51 |
| MIR1913 | 149,39 |
| MIR1205 | 149,19 |
| MIR548J | 148,88 |
| MIR4289 | 147,14 |
| MIR3928 | 146,29 |
| MIR4312 | 143,44 |
| MIR592 | 143,35 |
| MIR2114 | 142,09 |
| MIR1288 | 141,26 |
| MIR4681 | 140,73 |
| MIR26B | 139,54 |
| MIR3189 | 138,32 |
| MIR647 | 138,2 |
| MIR139 | 137,99 |
| MIR4497 | 137,83 |
| MIR4257 | 137,43 |
| MIR4665 | 136,65 |
| MIR5188 | 134,19 |
| MIR4493 | 133,07 |
| MIR4714 | 133 |
| MIR4730 | 130,42 |
| MIR4266 | 129,99 |
| MIR4486 | 129,98 |
| MIR548Z | 128,79 |
| MIR190A | 128,09 |
| MIR4671 | 127,11 |
| MIR483 | 125,35 |
| MIR3143 | 125,18 |
| MIR548AR | 124,64 |
| MIR3138 | 123,06 |
| MIR99B | 120,95 |
| MIR2682 | 120,61 |
| MIR3189 | 120,33 |
| MIR548N | 120,24 |
| MIR4316 | 119,97 |
| MIR1469 | 119,94 |
| MIR548A2 | 119,27 |
| MIR326 | 118,68 |
| MIR5188 | 118,64 |
| MIR29B1 | 118,27 |
| MIR3180-4 | 117,99 |
| MIR4269 | 117,47 |
| MIR657 | 117,34 |
| MIR34A | 116,23 |
| MIR4681 | 116,07 |
| MIR4257 | 116,05 |
| MIR3664 | 115,49 |
| MIR4265 | 114,8 |
| MIR4637 | 113,38 |
| MIR3654 | 113,33 |
| MIR301B | 111,78 |
| MIR3180-5 | 111,24 |
| MIR4683 | 110,26 |
| MIR614 | 109,67 |
| MIR3622A | 109,47 |
| MIR3202-2 | 109,46 |
| MIR3687 | 109,15 |
| MIR4523 | 108,19 |
| MIR612 | 107,8 |
| MIR4462 | 107,29 |
| MIR3679 | 106,76 |
| MIR3692 | 106,7 |
| MIR1244-2 | 106,61 |
| MIR4729 | 106,33 |
| MIR3142 | 105,84 |
| MIR4253 | 105,74 |
| MIR648 | 105,44 |
| MIR3143 | 105,27 |
| MIR4785 | 105,18 |
| MIR5188 | 104,52 |
| MIR548N | 104,11 |
| MIR138-2 | 103,83 |
| MIR3198-2 | 103,54 |
| MIR2467 | 103,46 |
| MIR3154 | 102,61 |
| MIR23A | 102,24 |
| MIR4530 | 101,81 |
| MIR3679 | 101,29 |
| MIR1471 | 101,09 |
| MIR3194 | 100,68 |
| MIR3188 | 99,51 |
| MIR139 | 99,25 |
| MIR3194 | 98,83 |
| MIR648 | 98,45 |
| MIR593 | 97,99 |
| MIR4691 | 97,84 |
| MIR2278 | 97,7 |
| MIR33B | 97,57 |
| MIR378D1 | 97,52 |
| MIR148A | 97,3 |
| MIR650 | 97,1 |
| MIR3193 | 97,07 |
| MIR624 | 96,58 |
| MIR4442 | 96,49 |
| MIR4761 | 95,95 |
| MIR634 | 95,67 |
| MIR612 | 95,16 |
| MIR3675 | 94,87 |
| MIR4681 | 94,77 |
| MIR34A | 94,55 |
| MIR3155B | 93,95 |
| MIR4304 | 93,21 |
| MIR4746 | 93,21 |
| MIR4262 | 93,03 |
| MIR9-3 | 92,9 |
| MIRLET7B | 92,48 |
| MIR4530 | 92,38 |
| MIR4284 | 92,35 |
| MIR4710 | 91,74 |
| MIR3679 | 91,39 |
| MIR34A | 91 |
| MIR4441 | 90,87 |
| MIR183 | 90,79 |
| MIR4462 | 90,76 |
| MIR34A | 90,75 |
| MIR3193 | 90,36 |
| MIR603 | 90,11 |
| MIR4766 | 90,1 |
| MIR217 | 90,01 |
| MIR4692 | 89,84 |
| MIR1179 | 89,73 |
| MIR126 | 89,49 |
| MIR5189 | 89,36 |
| MIR126 | 89,34 |
| MIR4655 | 89,26 |
| MIR4269 | 88,93 |
| MIR133A2 | 88,77 |
| MIR1284 | 88,04 |
| MIR4681 | 87,99 |
| MIR4708 | 87,58 |
| MIR548O2 | 86,8 |
| MIR548Z | 85,68 |
| MIR4777 | 85,32 |
| MIR373 | 84,89 |
| MIR648 | 84,82 |
| MIR598 | 84,29 |
| MIR4669 | 84,07 |
| MIR5091 | 84,07 |
| MIR3973 | 83,98 |
| MIR4668 | 83,84 |
| MIR3622B | 83,66 |
| MIR3138 | 83,39 |
| MIR1538 | 83,17 |
| MIR128-2 | 83,02 |
| MIR4786 | 82,78 |
| MIR5192 | 82,68 |
| MIR5703 | 82,68 |
| MIR661 | 82,63 |
| MIR3619 | 82,38 |
| MIR626 | 82,14 |
| MIR3675 | 81,95 |
| MIR1182 | 81,9 |
| MIR3187 | 81,76 |
| MIR1281 | 81,61 |
| MIR1206 | 81,56 |
| MIR152 | 81,52 |
| MIR5694 | 81,43 |
| MIR638 | 81,11 |
| MIR4441 | 81,09 |
| MIR1913 | 80,77 |
| MIR23A | 80,44 |
| MIR4634 | 80,42 |
| MIR155 | 80,35 |
| MIR1249 | 80,34 |
| MIR648 | 80,01 |
| MIR936 | 79,64 |
| MIR145 | 79,58 |
| MIR4734 | 79,58 |
| MIR5694 | 79,51 |
| MIR4440 | 79,38 |
| MIR4486 | 79,2 |
| MIR1281 | 79,02 |
| MIR639 | 78,94 |
| MIR4681 | 78,66 |
| MIR638 | 78,6 |
| MIR4767 | 78,41 |
| MIR598 | 78,41 |
| MIR4308 | 78,25 |
| MIR4743 | 78,22 |
| MIR578 | 78,18 |
| MIR101-1 | 78,16 |
| MIR10B | 78,11 |
| MIRLET7A1 | 78,1 |
| MIR103B2 | 77,95 |
| MIR1471 | 77,94 |
| MIR2114 | 77,94 |
| MIR4786 | 77,91 |
| MIR769 | 77,81 |
| MIR3622B | 77,56 |
| MIR1292 | 77,29 |
| MIR4708 | 77,07 |
| MIR101-1 | 76,68 |
| MIR548K | 76,6 |
| MIR149 | 76,57 |
| MIR3687 | 76,46 |
| MIR187 | 76,21 |
| MIR4490 | 76,15 |
| MIR1234 | 76,13 |
| MIR5580 | 75,98 |
| MIR130A | 75,8 |
| MIR1265 | 75,74 |
| MIR1265 | 75,7 |
| MIR4486 | 75,56 |
| MIR5192 | 75,25 |
| MIR1976 | 75,24 |
| MIR548AI | 75,2 |
| MIR4681 | 75,09 |
| MIR4696 | 74,89 |
| MIR3648 | 74,66 |
| MIR648 | 74,36 |
| MIRLET7I | 74,28 |
| MIR4319 | 74,14 |
| MIR548I4 | 74,1 |
| MIR1301 | 74,04 |
| MIR4669 | 73,85 |
| MIR34A | 73,68 |
| MIR4297 | 73,43 |
| MIR4475 | 73,42 |
| MIR4422 | 73,4 |
| MIR483 | 73,26 |
| MIR21 | 73,03 |
| MIR1284 | 73,01 |
| MIR4748 | 72,8 |
| MIR1226 | 72,65 |
| MIR103B2 | 72,56 |
| MIR548Z | 72,56 |
| MIR575 | 72,56 |
| MIR3201 | 72,4 |
| MIR4777 | 72,36 |
| MIR708 | 72,24 |
| MIR4636 | 72,16 |
| MIR4644 | 72,04 |
| MIR4478 | 72,01 |
| MIR4422 | 71,81 |
| MIR4766 | 71,81 |
| MIR4684 | 71,78 |
| MIR4666A | 71,63 |
| MIR548Z | 71,53 |
| MIR4786 | 71,52 |
| MIR129-2 | 71,37 |
| MIR2114 | 71,36 |
| MIR4693 | 71,15 |
| MIR885 | 70,69 |
| MIR3910-2 | 70,64 |
| MIR643 | 70,55 |
| MIR199A1 | 70,47 |
| MIR630 | 70,35 |
| MIR4693 | 70,34 |
| MIR4669 | 70,31 |
| MIR3199-1 | 70,25 |
| MIR629 | 70,06 |
| MIR4740 | 69,98 |
| MIR874 | 69,95 |
| MIR4472-2 | 69,89 |
| MIR1471 | 69,66 |
| MIR5700 | 69,63 |
| MIR4266 | 69,59 |
| MIR455 | 69,54 |
| MIR130A | 69,48 |
| MIR558 | 69,43 |
| MIR3664 | 69,32 |
| MIR4516 | 69,28 |
| MIR3648 | 69,21 |
| MIR4786 | 69,01 |
| MIR635 | 68,91 |
| MIR4635 | 68,75 |
| MIR4765 | 68,75 |
| MIR589 | 68,57 |
| MIR4282 | 68,37 |
| MIR1265 | 68,32 |
| MIR570 | 68,14 |
| MIR1256 | 67,97 |
| MIR5002 | 67,86 |
| MIR1246 | 67,83 |
| MIR4786 | 67,78 |
| MIR4266 | 67,75 |
| MIR657 | 67,75 |
| MIR483 | 67,66 |
| MIR5696 | 67,61 |
| MIR4747 | 67,57 |
| MIR3182 | 67,54 |
| MIR3162 | 67,32 |
| MIR330 | 67,15 |
| MIR548AR | 67,13 |
| MIR1237 | 66,97 |
| MIR326 | 66,94 |
| MIR5699 | 66,89 |
| MIR1287 | 66,83 |
| MIR3200 | 66,79 |
| MIR346 | 66,57 |
| MIR4254 | 66,43 |
| MIR23B | 66,28 |
| MIR4692 | 66,27 |
| MIR4266 | 66,11 |
| MIR4440 | 66,1 |
| MIR1227 | 66,09 |
| MIR378C | 65,99 |
| MIR4669 | 65,9 |
| MIR153-2 | 65,86 |
| MIR219-2 | 65,86 |
| MIR5699 | 65,85 |
| MIR29B2 | 65,82 |
| MIR4254 | 65,73 |
| MIR1226 | 65,7 |
| MIR202 | 65,7 |
| MIR3194 | 65,47 |
| MIR4648 | 65,47 |
| MIR3691 | 65,4 |
| MIR634 | 65,4 |
| MIR3074 | 65,39 |
| MIR598 | 65,38 |
| MIR3664 | 65,28 |
| MIR3614 | 65,16 |
| MIR2278 | 65,12 |
| MIR3165 | 65,09 |
| MIR378F | 65,04 |
| MIR4718 | 65,04 |
| MIR301A | 65 |
| MIR5100 | 64,96 |
| MIR5187 | 64,87 |
| MIR4485 | 64,73 |
| MIR3938 | 64,69 |
| MIR1244-2 | 64,57 |
| MIR3177 | 64,54 |
| MIR3654 | 64,54 |
| MIR3138 | 64,49 |
| MIR3134 | 64,39 |
| MIR4767 | 64,29 |
| MIR1301 | 64,24 |
| MIR4532 | 64,16 |
| MIR1284 | 64,08 |
| MIR3187 | 64,01 |
| MIR3122 | 63,88 |
| MIR3150B | 63,88 |
| MIR4681 | 63,81 |
| MIR3165 | 63,76 |
| MIR190A | 63,62 |
| MIR593 | 63,6 |
| MIR514A2 | 63,59 |
| MIR326 | 63,51 |
| MIR4636 | 63,5 |
| MIR641 | 63,33 |
| MIR4681 | 63,3 |
| MIR4309 | 63,22 |
| MIR4440 | 63,22 |
| MIR5194 | 63,22 |
| MIR4674 | 63,14 |
| MIR4786 | 63,12 |
| MIR3648 | 63,03 |
| MIR4532 | 62,98 |
| MIR4462 | 62,97 |
| MIRLET7A3 | 62,89 |
| MIR548AO | 62,88 |
| MIR4696 | 62,61 |
| MIR620 | 62,49 |
| MIR1908 | 62,48 |
| MIR4439 | 62,48 |
| MIR145 | 62,44 |
| MIR4417 | 62,44 |
| MIR3621 | 62,38 |
| MIR3619 | 62,34 |
| MIR4739 | 62,31 |
| MIR4714 | 62,27 |
| MIRLET7B | 62,24 |
| MIR1228 | 62,17 |
| MIR548T | 62,02 |
| MIR4757 | 61,91 |
| MIR4533 | 61,79 |
| MIR4513 | 61,74 |
| MIR143 | 61,63 |
| MIR3162 | 61,58 |
| MIR4749 | 61,49 |
| MIR222 | 61,43 |
| MIR492 | 61,4 |
| MIR4266 | 61,32 |
| MIR4735 | 61,24 |
| MIR634 | 61,24 |
| MIR499A | 61,17 |
| MIR2116 | 61,13 |
| MIR145 | 61,06 |
| MIR1915 | 61,01 |
| MIR26B | 60,95 |
| MIR4792 | 60,81 |
| MIR4790 | 60,8 |
| MIR4479 | 60,78 |
| MIR3687 | 60,7 |
| MIR3186 | 60,63 |
| MIR5093 | 60,55 |
| MIR29A | 60,4 |
| MIR3185 | 60,26 |
| MIR548K | 60,18 |
| MIR5093 | 60,1 |
| MIR4261 | 60,02 |
| MIR5189 | 59,98 |
| MIR4777 | 59,89 |
| MIR3687 | 59,86 |
| MIR4486 | 59,83 |
| MIR601 | 59,72 |
| MIR378D2 | 59,71 |
| MIR1204 | 59,65 |
| MIR4304 | 59,64 |
| MIR3973 | 59,49 |
| MIR596 | 59,48 |
| MIR22 | 59,47 |
| MIR1973 | 59,38 |
| MIR548X | 59,38 |
| MIR708 | 59,21 |
| MIR4706 | 59,14 |
| MIR3140 | 59,08 |
| MIR139 | 59,01 |
| MIR3620 | 59 |
| MIR191 | 58,98 |
| MIR1237 | 58,96 |
| MIR3200 | 58,91 |
| MIR891A | 58,83 |
| MIR4488 | 58,8 |
| MIR5700 | 58,8 |
| MIR3926-2 | 58,77 |
| MIR4648 | 58,71 |
| MIR221 | 58,65 |
| MIR3675 | 58,62 |
| MIR5092 | 58,62 |
| MIR4672 | 58,53 |
| MIR5192 | 58,53 |
| MIR4783 | 58,4 |
| MIR483 | 58,35 |
| MIR129-2 | 58,33 |
| MIR4440 | 58,28 |
| MIR3664 | 58,26 |
| MIR4689 | 58,26 |
| MIR34A | 58,17 |
| MIR150 | 58,1 |
| MIR5004 | 58,1 |
| MIR570 | 58,03 |
| MIR5010 | 57,97 |
| MIR573 | 57,93 |
| MIR3675 | 57,9 |
| MIR4285 | 57,9 |
| MIR4432 | 57,87 |
| MIR1289-2 | 57,82 |
| MIR630 | 57,76 |
| MIR551A | 57,71 |
| MIR4689 | 57,65 |
| MIR5191 | 57,63 |
| MIR4279 | 57,58 |
| MIR645 | 57,58 |
| MIR1284 | 57,52 |
| MIR3201 | 57,51 |
| MIR2117 | 57,47 |
| MIR3922 | 57,47 |
| MIR1289-2 | 57,39 |
| MIR4744 | 57,34 |
| MIR3132 | 57,33 |
| MIR3689C | 57,29 |
| MIR3689C | 57,26 |
| MIR4476 | 57,26 |
| MIR3202-2 | 57,24 |
| MIR4265 | 57,24 |
| MIR4505 | 57,16 |
| MIR4785 | 57,11 |
| MIR3651 | 57,08 |
| MIR4316 | 57,05 |
| MIR3141 | 56,93 |
| MIR4655 | 56,91 |
| MIR4710 | 56,86 |
| MIR1208 | 56,84 |
| MIR708 | 56,81 |
| MIR548AR | 56,79 |
| MIR5708 | 56,79 |
| MIR126 | 56,71 |
| MIR593 | 56,7 |
| MIR4508 | 56,62 |
| MIR1914 | 56,61 |
| MIR1226 | 56,57 |
| MIR30D | 56,56 |
| MIR4281 | 56,55 |
| MIR1322 | 56,45 |
| MIR4266 | 56,4 |
| MIR614 | 56,39 |
| MIR196A1 | 56,36 |
| MIR4798 | 56,29 |
| MIR365B | 56,24 |
| MIR548I4 | 56,17 |
| MIR4696 | 56,13 |
| MIR4655 | 56,12 |
| MIR1915 | 56,09 |
| MIR4735 | 56,08 |
| MIR505 | 56,02 |
| MIR3138 | 55,95 |
| MIR4292 | 55,95 |
| MIR644A | 55,88 |
| MIR4532 | 55,82 |
| MIR4505 | 55,79 |
| MIR557 | 55,72 |
| MIR4330 | 55,68 |
| MIR365A | 55,58 |
| MIR491 | 55,54 |
| MIR611 | 55,5 |
| MIR4441 | 55,49 |
| MIR1178 | 55,43 |
| MIR4261 | 55,43 |
| MIR548K | 55,34 |
| MIR548AR | 55,31 |
| MIR4266 | 55,28 |
| MIR5192 | 55,28 |
| MIR4466 | 55,22 |
| MIR218-2 | 55,06 |
| MIR1913 | 54,99 |
| MIR5189 | 54,97 |
| MIR210 | 54,92 |
| MIR4280 | 54,92 |
| MIR4297 | 54,92 |
| MIR10A | 54,88 |
| MIR4717 | 54,88 |
| MIR4744 | 54,88 |
| MIR4275 | 54,84 |
| MIR4744 | 54,82 |
| MIR2114 | 54,77 |
| MIR3194 | 54,77 |
| MIR326 | 54,77 |
| MIR4690 | 54,77 |
| MIR4472-1 | 54,75 |
| MIR3125 | 54,72 |
| MIR611 | 54,7 |
| MIR4274 | 54,68 |
| MIR4757 | 54,66 |
| MIR573 | 54,5 |
| MIR4486 | 54,46 |
| MIR3679 | 54,41 |
| MIR4701 | 54,4 |
| MIR2053 | 54,39 |
| MIR3177 | 54,29 |
| MIR4436A | 54,27 |
| MIR103B2 | 54,2 |
| MIR139 | 54,2 |
| MIR4317 | 54,17 |
| MIR4436B1 | 54,17 |
| MIR657 | 54,13 |
| MIR548AR | 54,09 |
| MIR4440 | 54,08 |
| MIR3605 | 54,06 |
| MIR3910-2 | 54,06 |
| MIR4531 | 54,06 |
| MIR3664 | 54,05 |
| MIR326 | 54,01 |
| MIR4681 | 54,01 |
| MIR3664 | 53,98 |
| MIR4489 | 53,98 |
| MIR641 | 53,98 |
| MIR4682 | 53,93 |
| MIR4706 | 53,91 |
| MIR5584 | 53,91 |
| MIR3691 | 53,88 |
| MIR629 | 53,77 |
| MIR3122 | 53,74 |
| MIR3138 | 53,7 |
| MIR4486 | 53,7 |
| MIR4696 | 53,7 |
| MIR5002 | 53,7 |
| MIR4668 | 53,67 |
| MIR143 | 53,63 |
| MIR1227 | 53,56 |
| MIR3201 | 53,56 |
| MIR3659 | 53,56 |
| MIR5093 | 53,56 |
| MIR3909 | 53,55 |
| MIR221 | 53,54 |
| MIR548AN | 53,54 |
| MIR4262 | 53,49 |
| MIR4441 | 53,49 |
| MIR3123 | 53,45 |
| MIR1289-2 | 53,42 |
| MIR4444-1 | 53,42 |
| MIR4681 | 53,42 |
| MIR4735 | 53,4 |
| MIR3202-2 | 53,39 |
| MIR1913 | 53,29 |
| MIR181B2 | 53,28 |
| MIR4642 | 53,28 |
| MIR139 | 53,27 |
| MIR190A | 53,26 |
| MIR345 | 53,23 |
| MIR1206 | 53,21 |
| MIR153-2 | 53,21 |
| MIR3689C | 53,21 |
| MIR4743 | 53,21 |
| MIR4420 | 53,15 |
| MIR153-1 | 53,12 |
| MIR1205 | 53,07 |
| MIR3664 | 53,07 |
| MIR378C | 53,07 |
| MIR4632 | 53,07 |
| MIR650 | 53,07 |
| MIR211 | 53,04 |
| MIR4493 | 53,03 |
| MIR4505 | 53,03 |
| MIR641 | 53 |
| MIR3194 | 52,94 |
| MIR34A | 52,94 |
| MIR874 | 52,94 |
| MIR5008 | 52,92 |
| MIR3154 | 52,87 |
| MIR1228 | 52,84 |
| MIR4265 | 52,8 |
| MIR602 | 52,77 |
| MIR3155B | 52,74 |
| MIR4522 | 52,71 |
| MIR340 | 52,69 |
| MIR4296 | 52,69 |
| MIR2964A | 52,62 |
| MIR3658 | 52,62 |
| MIR601 | 52,54 |
| MIR4480 | 52,52 |
| MIR4787 | 52,52 |
| MIR128-2 | 52,51 |
| MIR2682 | 52,49 |
| MIR4265 | 52,44 |
| MIR1537 | 52,43 |
| MIR326 | 52,42 |
| MIR3922 | 52,41 |
| MIR548D2 | 52,41 |
| MIR1915 | 52,39 |
| MIR5699 | 52,39 |
| MIR5092 | 52,36 |
| MIR4266 | 52,25 |
| MIR4289 | 52,25 |
| MIR4694 | 52,22 |
| MIR4718 | 52,18 |
| MIR4802 | 51,95 |
| MIR4510 | 51,94 |
| MIR5093 | 51,87 |
| MIR551A | 51,87 |
| MIR218-2 | 51,85 |
| MIR3188 | 51,79 |
| MIR4446 | 51,79 |
| MIR593 | 51,78 |
| MIR5193 | 51,72 |
| MIR3156-2 | 51,65 |
| MIR3663 | 51,58 |
| MIR5189 | 51,58 |
| MIR604 | 51,57 |
| MIR3194 | 51,5 |
| MIR4743 | 51,44 |
| MIR4505 | 51,42 |
| MIR3661 | 51,41 |
| MIR23B | 51,35 |
| MIR3155A | 51,35 |
| MIR4743 | 51,35 |
| MIR5188 | 51,35 |
| MIR219-1 | 51,32 |
| MIR4659B | 51,26 |
| MIR4297 | 51,25 |
| MIR4694 | 51,23 |
| MIR4710 | 51,18 |
| MIR4753 | 51,17 |
| MIR1976 | 51,16 |
| MIR4799 | 51,16 |
| MIR548K | 51,15 |
| MIR1275 | 51,13 |
| MIR4632 | 51,13 |
| MIR4744 | 51,12 |
| MIR1260B | 51,1 |
| MIR149 | 51,08 |
| MIR4310 | 51,06 |
| MIR4656 | 50,99 |
| MIR4714 | 50,96 |
| MIR612 | 50,94 |
| MIR4739 | 50,92 |
| MIR4799 | 50,92 |
| MIR2277 | 50,91 |
| MIR5100 | 50,86 |
| MIR3664 | 50,84 |
| MIR4757 | 50,84 |
| MIR635 | 50,83 |
| MIR4684 | 50,77 |
| MIR598 | 50,77 |
| MIR548K | 50,75 |
| MIR4493 | 50,7 |
| MIR3620 | 50,63 |
| MIR99A | 50,63 |
| MIR133A2 | 50,6 |
| MIR4486 | 50,59 |
| MIR612 | 50,59 |
| MIR620 | 50,59 |
| MIR4472-2 | 50,56 |
| MIR1537 | 50,51 |
| MIR4462 | 50,48 |
| MIR593 | 50,48 |
| MIR3922 | 50,45 |
| MIR935 | 50,45 |
| MIR200B | 50,39 |
| MIR135A1 | 50,34 |
| MIR455 | 50,34 |
| MIR601 | 50,34 |
| MIR92B | 50,27 |
| MIR3200 | 50,21 |
| MIR5008 | 50,21 |
| MIR5093 | 50,2 |
| MIR4262 | 50,17 |
| MIR4531 | 50,13 |
| MIR4648 | 50,13 |
| MIR4261 | 50,08 |
| MIR3194 | 50,06 |
| MIR4674 | 50,06 |
| MIR4740 | 50,06 |
| MIR3924 | 50,05 |
| MIR670 | 50,04 |
| MIR326 | 50,02 |
| MIR548W | 50,02 |

**References in File S1.**

1. Serchov T, Dubois-Pot-Schneider H, Charlot C, Rosl F, et al. (2010) Involvement of net and Hifα in distinct yet intricately linked hypoxia-induced signaling pathways. Journal of Biological Chemistry 285: 21223-21232.

2. Kulshreshtha R, Ferracin M, Wojcik SE, Garzon R, Alder H, et al. (2007) A microRNA signature of hypoxia. Molecular and Cellular Biology 27: 1859-1867.

3. Shen G, Li X, Jia YF, Piazza GA, Xi Y (2013) Hypoxia-regulated microRNAs in human cancer. Acta Pharmacologica Sinica 34: 336-341.
