# Supplementary figures and images for: The Oncogenic MicroRNA Hsa-miR-155-5p Targets the Transcription Factor ELK3 and Links It to the Hypoxia Response
Source: PLoS One. 2014 Nov 17;9(11):e113050. doi: 10.1371/journal.pone.0113050 (PMC4234625; doi:10.1371/journal.pone.0113050)

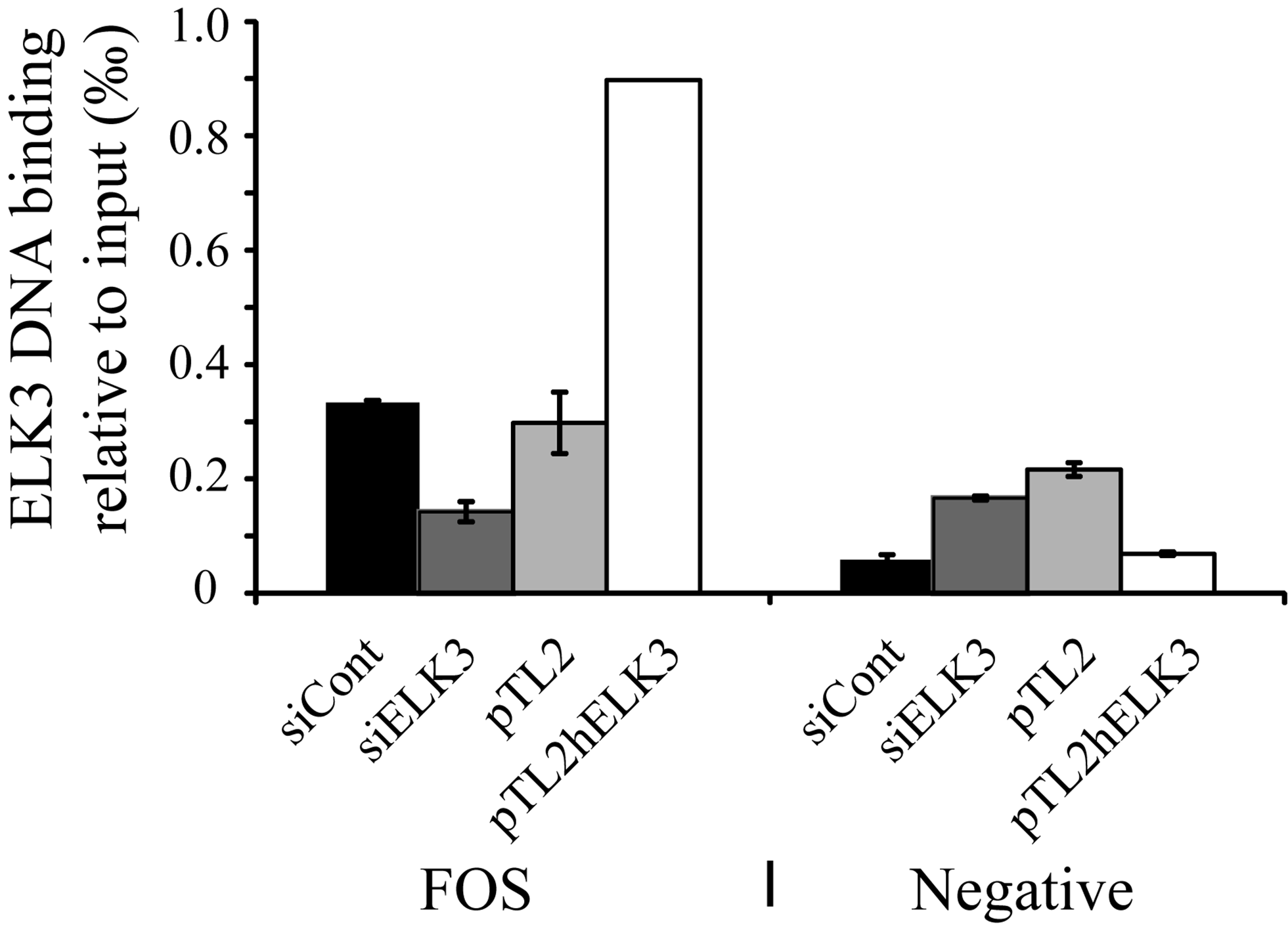

Supplement: Figure S1 — Validation of the ELK3 antibody for ChIP-seq. (A) ChIP on HUVEC cells transfected with siRNA against ELK3 (siELK3) or overexpression plasmid (pTL2hELK3) and the corresponding controls (siCont and pTL2). DNA in the immunoprecipitates were analysed by QPCR with primers for a region in the c-FOS promoter and a negative control DNA region expected not to bind ELK3. (TIF) [file pone.0113050.s002.tif]

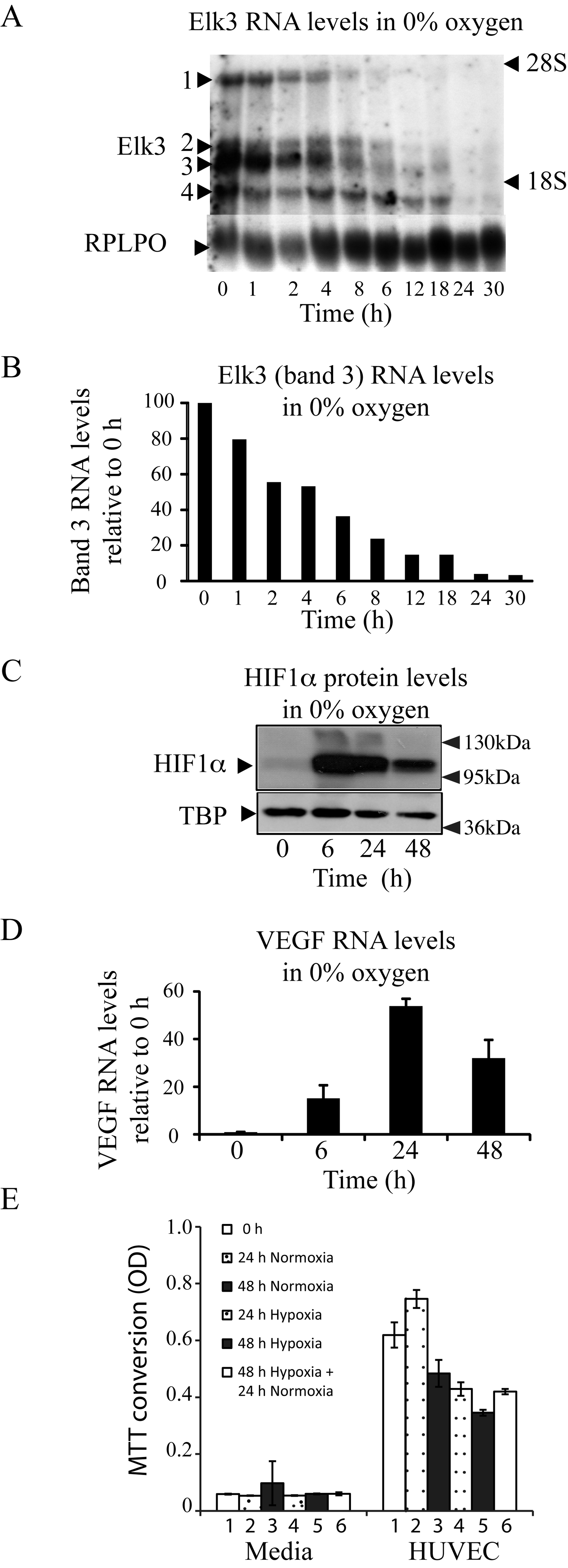

Supplement: Figure S2 — ELK3 mRNA degradation in anoxia (0% oxygen) in SEND cells. (A) Northern blot of total RNA extracted from SEND cells cultured in 0% oxygen for the designated times and probed for ELK3 and RPLPO. The locations of 18S and 28S ribosomal RNA are indicated. (B) Northern Blot quantification. The major Elk3 band (3, 1,600 bases) was quantified by scanning suitably exposed autoradiograms, correcting for RPLPO, and normalizing to the zero time point. One representative example of four independent experiments is shown. (C) HIF1α induction under 0% oxygen conditions. HUVEC cells were incubated in 0% oxygen for the indicated times and whole cell extracts were analysed by western blotting with antibodies against HIF1α and TBP as a loading control. (D) VEGF mRNA induction under 0% oxygen conditions. HUVEC cells were incubated in 0% oxygen for the indicated times, and extracted RNA was analysed by RT-QPCR for VEGF and RPLPO RNA levels. VEGF RNA levels were corrected for the internal control RPLPO, and normalized to the 0 h time point. (E) HUVEC cells viability during hypoxia in a MTT assay in comparison to media without cells. Measurements were taken at the start of the experiment (lane 1) and after 24 and 48 hours of normoxia (lanes 2 and 3, respectively) or hypoxia (lanes 4 and 5, respsctively). A further point was taken after 24 hours of re-oxygenation (lanes 6). Error bars represent the S.D. within the experiment. (TIF) [file pone.0113050.s003.tif]
